# Supplementary material for: The prevalence of respectful maternity care during childbirth and its determinants in Ethiopia: A systematic review and meta-analysis
Source: PLoS One. 2022 Nov 23;17(11):e0277889. doi: 10.1371/journal.pone.0277889 (PMC9683616; doi:10.1371/journal.pone.0277889)
Supplement: S2 File — (DOCX) [file pone.0277889.s002.docx]

**S2File: Examples of searching strategy for systematic review and meta-analysis on Respectful maternity care and its determinants in Ethiopia, 2022**

| **Database** | **Example of searching strategy** | **Number of studies** |
| --- | --- | --- |
| PubMed | (((((((((((((("Respectful"[tw]) OR ("Woman-Centered"[tw])) OR ("Dignified"[tw])) OR ("Friendly"[tw])) OR ("non-Abusive"[tw])) OR ("Compassionate"[tw])) OR ("non-discriminatory"[tw])) AND ("maternity care"[tw])) OR ("maternal health care"[tw])) OR ("Prenatal care"[tw])) OR ("Antenatal care"[tw])) OR ("Postnatal care"[tw])) OR ("postpartum Care"[tw])) OR ("Maternal Care"[tw])) AND ("Ethiopia"[tw]) | 1231 |
| Google Scholar | allintitle: Ethiopia respectful OR compassionate OR non-abusive "care" | 59 |
| Science Direct | "Respectful maternity care" AND "Ethiopia" | 37 |
| Scopus | TITLE-ABS-KEY ( '' Respectful maternity care'' AND Ethiopia | 48 |
| ProQuest | "Respectful maternity care" AND "Ethiopia" | 135 |
| Web of science | TOPIC: (Respectful maternity care and Ethiopia) | 24 |
| Cochrane Library | ("Respectful maternity care” OR "non-abusive maternal care) AND Ethiopia | 22 |
| Direct of Open Access Journals | “Respectful maternity care” AND "Ethiopia” | 37 |
