# Supplementary material for: The prevalence of respectful maternity care during childbirth and its determinants in Ethiopia: A systematic review and meta-analysis
Source: PLoS One. 2022 Nov 23;17(11):e0277889. doi: 10.1371/journal.pone.0277889 (PMC9683616; doi:10.1371/journal.pone.0277889)
Supplement: S3 File — (DOCX) [file pone.0277889.s003.docx]

**S3File:** JBI Critical Appraisal Checklist for prevalence studies used for assessing the individual quality of 16 studies included in the systematic review and meta-analysis, 2022.

| Author name, Year of Publication | The sampling frame appropriate to address the target population | The study subjects and the setting described in detail | Study participants sampled in an appropriate way | Study subjects and the setting described in detail | Data analysis conducted with sufficient coverage of the identified sample | Valid methods used for the identification of the condition | Reliability of the instrument used to measure the condition | Appropriateness of Statistical analysis methods | Adequacy and management of Response rate | Total | Risk of bias |
| --- | --- | --- | --- | --- | --- | --- | --- | --- | --- | --- | --- |
| Amsalu et al., 2022 | 0 | 0 | 0 | 0 | 0 | 1 | 1 | 0 | 0 | 2 | Low |
| Yismaw et al., 2022 | 0 | 0 | 0 | 0 | 0 | 1 | 1 | 0 | 0 | 2 | Low |
| Yalew et al., 2022 | 0 | 0 | 0 | 0 | 0 | 1 | 1 | 0 | 0 | 2 | Low |
| Eneyew et al., 2021 | 0 | 0 | 0 | 1 | 1 | 0 | 1 | 0 | 0 | 3 | Moderate |
| Adane et al., 2021 | 0 | 0 | 0 | 0 | 0 | 1 | 0 | 0 | 0 | 1 | Low |
| Ambachew, 2021 | 0 | 0 | 0 | 0 | 0 | 1 | 1 | 0 | 0 | 2 | Low |
| Cafo et al., 2021 | 0 | 0 | 0 | 0 | 0 | 1 | 0 | 0 | 0 | 1 | Low |
| Abdo et al., 2021 | 0 | 0 | 0 | 1 | 0 | 1 | 1 | 0 | 0 | 3 | Moderate |
| Wochefu et al, 2021 | 0 | 0 | 0 | 0 | 0 | 1 | 1 | 0 | 0 | 2 | Low |
| Bante et al., 2020 | 0 | 0 | 0 | 0 | 0 | 1 | 0 | 0 | 0 | 1 | Low |
| Bulto et al., 2020 | 0 | 0 | 0 | 0 | 0 | 1 | 1 | 0 | 0 | 2 | Low |
| Yosef et al., 2020 | 0 | 0 | 0 | 0 | 0 | 1 | 1 | 0 | 0 | 2 | Low |
| Wubetu et al., 2020 | 0 | 0 | 0 | 0 | 0 | 1 | 1 | 0 | 0 | 2 | Low |
| Dagnaw et al.,2020 | 0 | 0 | 0 | 0 | 0 | 1 | 0 | 0 | 0 | 1 | Low |
| Wassihun and Zeleke, 2018 | 0 | 0 | 0 | 0 | 0 | 0 | 1 | 0 | 0 | 1 | Low |
| Asefa and Bekele, 2015 | 0 | 0 | 0 | 0 | 0 | 0 | 0 | 0 | 0 | 2 | Low |
