# Supplementary material for: The prevalence of respectful maternity care during childbirth and its determinants in Ethiopia: A systematic review and meta-analysis
Source: PLoS One. 2022 Nov 23;17(11):e0277889. doi: 10.1371/journal.pone.0277889 (PMC9683616; doi:10.1371/journal.pone.0277889)
Supplement: Attachment — Submitted filename: PONE-D-22-19516.pdf [file pone.0277889.s006.pdf]

PLOS ONE

Towards the quality of maternal and newborn health care: The level and determinants of respectful maternity care during childbirth in Ethiopia: A systematic review and Meta-analysis

--Manuscript Draft--

**Manuscript Number:**	PONE-D-22-19516
**Article Type:**	Research Article
**Full Title:**	Towards the quality of maternal and newborn health care: The level and determinants of respectful maternity care during childbirth in Ethiopia: A systematic review and Meta-analysis
**Short Title:**	The level and determinants of respectful maternity care in Ethiopia
**Corresponding Author:**	Aklilu Habte, MPH<br>Wachemo university<br>Hossana, ETHIOPIA
**Keywords:**	Maternity Care, Respectful maternity care, Determinants, Systematic review and meta-analysis, Ethiopia
**Abstract:**	Background:<br>Every woman has the right to woman-centered healthcare that is safe, effective, timely, respectful, and free of violence and discrimination during pregnancy, labor, and childbirth. Respectful maternity care (RMC) refers to care that includes the right to access friendly, abuse-free, timely, and discrimination-free maternal health care. Although several epidemiological studies on the magnitude and determinants of RMC in Ethiopia have been conducted, the results have been inconsistent and varied. This makes drawing equivocal conclusions and evidence at the national level harder. Hence, this systematic review and meta-analysis aimed at estimating the pooled prevalence of RMC and its determinants in Ethiopia.<br>Methods<br>Studies conducted until June 30, 2022, were searched by using PubMed, Google Scholar, Science Direct, Scopus, ProQuest, Web of Science, Cochrane Library, and Direct of Open Access Journals. Searching was carried out from May 15- June 30, 2022. In total, sixteen studies were considered in the final analysis. The data were extracted using Microsoft Excel and analyzed using STATA 16 software. The methodological quality of included studies was assessed by using Joanna Briggs Institute's critical appraisal checklist for prevalence studies. To estimate the pooled national prevalence of RMC, a random effect model with a DerSimonian Laired method was used. To assess the heterogeneity of the included studies, the Cochrane Q test statistics and I2 tests were used. To detect the presence of publication bias, a funnel plot and the Begg's and Egger's tests were used.<br>Results<br>Sixteen studies were eligible for this systematic review and meta-analysis with a total of 6354 study participants. The overall pooled prevalence of RMC in Ethiopia was 48.44% (95% CI: 39.02- 57.87). Receiving service by CRC-trained health care providers [AOR: 4.09, 95% CI: 1.73, 6.44], having ANC visits [AOR: 2.34, 95% CI: 1.62, 3.06], planning status of the pregnancy [AOR = 4.43, 95% CI: 2.74, 6.12], giving birth during the daytime [AOR: 2.61, 95% CI: 1.92, 3.31], and experiencing an obstetric complication[AOR: 0.46, 95% CI: 0.30, 0.61] were identified as determinants of RMC.<br>Conclusion:<br>As per this meta-analysis, the level of RMC in Ethiopia was low (48.44 percent), suggesting that more emphasis is needed to plan and implement intervention measures. Managers in the health sector should give due emphasis to the provision of CRC training for health care providers, who work at maternity service delivery points. Stakeholders need to work to increase the uptake of prenatal care to improve client-provider relationships across a continuum of care. Human resource managers should assign an adequate number of HCPs to the night-shift duties to reduce the workload on obstetric providers. A due emphasis should be given to those women who developed an obstetric complication through a continuous follow-up.
**Order of Authors:**	Aklilu Habte, MPH in Reproductive Health

Powered by Editorial Manager® and ProduXion Manager® from Aries Systems Corporation

	Aiggan Hailegebireal Tamene, MPH in Environmental health
	Demelash Woldeyohannes, Assistant Professor of Public Health
	Fitsum Endale, Msc in Human Nutrition
	Biruk Bogale, MPH in Epidemiology and Biostatstic
	Addisalem Gizachew, Msc in Human Nutrition
**Additional Information:**	
**Question**	**Response**
**Financial Disclosure**<br><br>Enter a financial disclosure statement that describes the sources of funding for the work included in this submission. Review the submission guidelines for detailed requirements. View published research articles from *PLOS ONE* for specific examples.<br><br>This statement is required for submission and **will appear in the published article** if the submission is accepted. Please make sure it is accurate.<br><br>**Unfunded studies**<br>Enter: *The author(s) received no specific funding for this work.*<br><br>**Funded studies**<br>Enter a statement with the following details:<br>• Initials of the authors who received each award<br>• Grant numbers awarded to each author<br>• The full name of each funder<br>• URL of each funder website<br>• Did the sponsors or funders play any role in the study design, data collection and analysis, decision to publish, or preparation of the manuscript?<br>• **NO** - Include this sentence at the end of your statement: *The funders had no role in study design, data collection and analysis, decision to publish, or preparation of the manuscript.*<br>• **YES** - Specify the role(s) played.<br><br>* typeset	The author(s) received no specific funding for this work.
**Competing Interests**<br><br>Use the instructions below to enter a competing interest statement for this submission. On behalf of all authors,	The authors have declared that no competing interests exist.

disclose any competing interests that could be perceived to bias this work—acknowledging all financial support and any other relevant financial or non-financial competing interests.<br><br>This statement is required for submission and **will appear in the published article** if the submission is accepted. Please make sure it is accurate and that any funding sources listed in your Funding Information later in the submission form are also declared in your Financial Disclosure statement.<br><br>View published research articles from *PLOS ONE* for specific examples.<br><br>**NO authors have competing interests**<br><br>Enter: *The authors have declared that no competing interests exist.*<br><br>**Authors with competing interests**<br><br>Enter competing interest details beginning with this statement:<br><br>*I have read the journal's policy and the authors of this manuscript have the following competing interests: [insert competing interests here]*<br><br>* typeset	
**Ethics Statement**<br><br>Enter an ethics statement for this submission. This statement is required if the study involved:<br><br>• Human participants<br>• Human specimens or tissue<br>• Vertebrate animals or cephalopods<br>• Vertebrate embryos or tissues<br>• Field research<br><br>Write "N/A" if the submission does not require an ethics statement.	N/A

Powered by Editorial Manager® and ProduXion Manager® from Aries Systems Corporation

General guidance is provided below. Consult the submission guidelines for detailed instructions. **Make sure that all information entered here is included in the Methods section of the manuscript.**<br><br>**Format for specific study types**<br><br>**Human Subject Research (involving human participants and/or tissue)**<br>• Give the name of the institutional review board or ethics committee that approved the study<br>• Include the approval number and/or a statement indicating approval of this research<br>• Indicate the form of consent obtained (written/oral) or the reason that consent was not obtained (e.g. the data were analyzed anonymously)<br><br>**Animal Research (involving vertebrate animals, embryos or tissues)**<br>• Provide the name of the Institutional Animal Care and Use Committee (IACUC) or other relevant ethics board that reviewed the study protocol, and indicate whether they approved this research or granted a formal waiver of ethical approval<br>• Include an approval number if one was obtained<br>• If the study involved *non-human primates*, add *additional details* about animal welfare and steps taken to ameliorate suffering<br>• If anesthesia, euthanasia, or any kind of animal sacrifice is part of the study, include briefly which substances and/or methods were applied<br><br>**Field Research**<br><br>Include the following details if this study involves the collection of plant, animal, or other materials from a natural setting:<br>• Field permit number<br>• Name of the institution or relevant body that granted permission	
**Data Availability**<br><br>Authors are required to make all data	Yes - all data are fully available without restriction

Powered by Editorial Manager® and ProduXion Manager® from Aries Systems Corporation

underlying the findings described fully available, without restriction, and from the time of publication. PLOS allows rare exceptions to address legal and ethical concerns. See the PLOS Data Policy and FAQ for detailed information.<br><br>A Data Availability Statement describing where the data can be found is required at submission. Your answers to this question constitute the Data Availability Statement and **will be published in the article**, if accepted.<br><br>**Important:** Stating 'data available on request from the author' is not sufficient. If your data are only available upon request, select 'No' for the first question and explain your exceptional situation in the text box.<br><br>Do the authors confirm that all data underlying the findings described in their manuscript are fully available without restriction?	
**Describe where the data may be found in full sentences. If you are copying our sample text, replace any instances of XXX with the appropriate details.**<br><br>• If the data are **held or will be held in a public repository**, include URLs, accession numbers or DOIs. If this information will only be available after acceptance, indicate this by ticking the box below. For example: *All XXX files are available from the XXX database (accession number(s) XXX, XXX.*).<br>• If the data are all contained **within the manuscript and/or Supporting Information files**, enter the following: *All relevant data are within the manuscript and its Supporting Information files.*<br>• If neither of these applies but you are able to provide **details of access elsewhere**, with or without limitations, please do so. For example:<br><br>*Data cannot be shared publicly because of [XXX]. Data are available from the*	All relevant data are within the manuscript and its Supporting Information files.

Powered by Editorial Manager® and ProduXion Manager® from Aries Systems Corporation

XXX Institutional Data Access / Ethics Committee (contact via XXX) for researchers who meet the criteria for access to confidential data.<br><br>*The data underlying the results presented in the study are available from (include the name of the third party and contact information or URL).*<br>• This text is appropriate if the data are owned by a third party and authors do not have permission to share the data.<br><br>* typeset	
Additional data availability information:	

Powered by Editorial Manager® and ProduXion Manager® from Aries Systems Corporation

Manuscript Click here to access/download;Manuscript;Manuscript.docx

Towards the quality of maternal and newborn health care: The level and determinants of respectful maternity care during childbirth in Ethiopia: A systematic review and Meta-analysis

Aklilu Habte[1]*, Aiggan Tamene[1], Demelash WoldeYohannes[1], Fitsum Endale[1], Biruk Bogale[2], Addisalem Gizachew[1]

[1]School of Public Health, College of Medicine and Health Sciences, Wachemo University, Hosanna, Ethiopia

[2]School of Public Health, College of Medicine and Health Sciences, Mizan-Tepi University, Mizan Aman, Ethiopia

[*] Corresponding author: akliluhabte57@gmail.com

Abstract

Background:

Every woman has the right to woman-centered healthcare that is safe, effective, timely, respectful, and free of violence and discrimination during pregnancy, labor, and childbirth. Respectful maternity care (RMC) refers to care that includes the right to access friendly, abuse-free, timely, and discrimination-free maternal health care. Although several epidemiological studies on the magnitude and determinants of RMC in Ethiopia have been conducted, the results have been inconsistent and varied. This makes drawing equivocal conclusions and evidence at the national level harder. Hence, this systematic review and meta-analysis aimed at estimating the pooled prevalence of RMC and its determinants in Ethiopia.

Methods

Studies conducted until June 30, 2022, were searched by using PubMed, Google Scholar, Science Direct, Scopus, ProQuest, Web of Science, Cochrane Library, and Direct of Open Access Journals. Searching was carried out from May 15- June 30, 2022. In total, sixteen studies were considered in the final analysis. The data were extracted using Microsoft Excel and analyzed using STATA 16 software. The methodological quality of included studies was assessed by using Joanna Briggs Institute's critical appraisal checklist for prevalence studies. To estimate the pooled national prevalence of RMC, a random effect model with a DerSimonian Laired method was used. To assess the heterogeneity of the included studies, the Cochrane Q test statistics and I2 tests were used. To detect the presence of publication bias, a funnel plot and the Begg's and Egger's tests were used.

Results

Sixteen studies were eligible for this systematic review and meta-analysis with a total of 6354 study participants. The overall pooled prevalence of RMC in Ethiopia was 48.44% (95% CI: 39.02- 57.87). Receiving service by CRC-trained health care providers [AOR: 4.09, 95% CI: 1.73, 6.44], having ANC visits [AOR: 2.34, 95% CI: 1.62, 3.06], planning status of the pregnancy [AOR = 4.43, 95% CI: 2.74, 6.12], giving birth during the daytime [AOR: 2.61, 95% CI: 1.92, 3.31], and experiencing an obstetric complication[AOR: 0.46, 95% CI: 0.30, 0.61] were identified as determinants of RMC.

Conclusion:

As per this meta-analysis, the level of RMC in Ethiopia was low (48.44 percent), suggesting that more emphasis is needed to plan and implement intervention measures. Managers in the health sector should give due emphasis to the provision of CRC training for health care

providers, who work at maternity service delivery points. Stakeholders need to work to increase the uptake of prenatal care to improve client-provider relationships across a continuum of care. Human resource managers should assign an adequate number of HCPs to the night-shift duties to reduce the workload on obstetric providers. A due emphasis should be given to those women who developed an obstetric complication through a continuous follow-up.

Introduction

Labor and, in particular, delivery are sensitive and vulnerable times in a woman's life[1]. Every woman has the right to woman-centered healthcare that is safe, effective, timely, respectful, and free of violence and discrimination during pregnancy, labor, and childbirth [2, 3]. Maternity care is a service that focused on improving maternal and newborn health outcomes during pregnancy, childbirth, and the postpartum period[4]. It includes monitoring the mother's and baby's well-being, health education, and assistance during childbirth[5].

Every year, around 140 million births occur worldwide, the vast majority of which are vaginal births with little difficulty for women and their newborns [6]. Pain, anxiety, threat, and exposure to the circumstance are the causes of women's vulnerability during labor and delivery [1]. Despite significant advances in maternal and child health, there is still a high rate of maternal and neonatal deaths globally[7]. Poor childbirth care contributes directly and indirectly to 82 percent of this problem[5]. Furthermore, even after reaching a health facility, many women and their babies die as a result of inadequate care[8]. Governments need to prioritize improving the quality of clinical and nonclinical care provided to women throughout pregnancy, delivery, and the early postpartum period to achieve the global maternal mortality ratio target of 70 per 100,000 live births by 2030 [3, 9].

Following mounting evidence of mistreatment of women during pregnancy and childbirth around the world, the WHO declared the prevention and elimination of disrespect and abuse during childbirth by implementing the Respectful Maternity Care (RMC) initiative[2, 10]. RMC is one of the WHO's eight dimensions for quality maternal and newborn health care, and it refers to care that includes the right to access friendly, abuse-free, timely, and discrimination-free maternal health care, along with privacy, confidentiality, equality, informed consent, and autonomy[11, 12]. It is a care that is respectful of and responsive to the preferences, needs, and values of individual women and their families[13]. It is a strategy that will be put in place to encourage positive interpersonal relationships between women and health care providers and workers throughout labor, delivery, and the postpartum period[14]. This notion advocates for

good staff attitudes, behaviors, and accountability that contribute to women's contentment with their birth experience in a sustainable way[14].

The concept of "safe motherhood" in delivery is limited to physical safety, although birth has significant cultural and personal meaning for both the women and their families[7]. Thus, this should extend beyond issues such as morbidity or mortality prevention to other concepts such as quality aspects by providing RMC[15].

Currently, the change from home delivery to hospital birth has increased access to lifesaving care for difficulties, but it has also generated new challenges, such as facility overcrowding, an excess of procedures, mistreatment, and over-medicalization [16]. Facility birth needs to automatically result in better outcomes in terms of quality, respectful, evidence-based care[16]. Such timely, respectful, and consensual obstetric care, however, is not the norm in many healthcare settings around the world, especially in developing countries like Ethiopia [16]. There is a widespread view that ensuring safe birth necessitates prioritizing the needs and objectives of health providers over the needs and priorities of childbearing women [14]. This creates and maintains a power imbalance, favoring providers and escalating to obstetric violence. Similar power dynamics amongst providers can cause unproductive and even toxic interactions between members of the care team, weakening the quality of care and resulting in provider dissatisfaction[17].

Poor interpersonal communication between client and provider during maternity care in low-resource settings like Ethiopia is increasingly being recognized as a barrier to getting skilled care for normal and complicated childbirth[18]. Despite a recent dramatic increase in the number of skilled providers and health facilities in Ethiopia, the uptake of prenatal care, skilled delivery service, and postnatal care remain at only 68%, 28%, and 17%, respectively[19]. Even though numerous circumstances contribute to low healthcare utilization, it is becoming evident that poor service quality and concerns about provider mistreatment are among the reasons why many women are unable to seek maternal, neonatal, and child health (MNCH) services[20]. Several studies have revealed that women's expectations of how they would be treated at health facilities may have a substantial impact on where they prefer to get maternal health services, notably childbirth [21-23].

In 2016, the Ethiopian government launched its Health Sector Transformation Plan, which aims to promote compassionate and respectful care (CRC), with an emphasis on RMC, to improve maternal and newborn health outcomes[24]. Although the target has not yet been met, this plan emphasizes the need of achieving 90 percent skilled birth attendance and lowering the maternal

mortality ratio (MMR) from 420/100,000 live births in 2015 to 199/100,000 live births by 2020[24]. Improving the quality of care through enhancing RMC has been highlighted as the most important method for lowering maternal and newborn mortality by laying the path for skilled delivery, particularly in developing countries like Ethiopia [5, 6]. Understanding the level and factors that contribute to RMC can help to improve the effectiveness of RMC initiatives and may have a beneficial impact on the uptake of MNCH services[25].

Although several epidemiological studies on the magnitude and determinants of RMC in Ethiopia have been conducted, the results have been inconsistent and varied. Furthermore, existing studies have also been small-scale or limited by locality, which might also make drawing equivocal conclusions and evidence at the national level harder. Furthermore, such disparities may be inadequate for policymakers and planners to intervene, demanding an assessment of the pooled estimates. Hence, this systematic review and meta-analysis aimed at estimating the pooled prevalence of RMC and its determinants at the national level. The study's findings will help policymakers and program planners build appropriate interventions to enhance the level of RMC, which is one of the four pillars of HSTP[24].

Methods

Study design

While conducting this systematic review and meta-analysis, the Preferred Reporting Items for Systematic Reviews and Meta-Analyses (PRISMA) standards for literature search method, study selection, data extraction, and result reporting were followed [26](**S1File**). To establish the inclusion and exclusion criteria, the PEO (Population, Exposure of interest, Outcome) technique was used, which was adapted from the JBI 2017 review guideline[27].

Eligibility criteria

Inclusion criteria

Population: Women in the reproductive age group (15-49)

Exposure of interest: Maternity care (prenatal, skilled delivery, and postnatal) cares

Outcome: Receiving respectful maternity care (RMC).

Study designs: All crossectional studies reporting the level of RMC and its determinants were considered.

Study setting: Only studies conducted in Ethiopia were considered.

Publication status: Both published and unpublished studies were considered, and if a study appeared in multiple reports, the most comprehensive and up-to-date one had been used.

Language: Articles published in the English language were considered.

Year of publication: All publications reported before June 30, 2022, were taken into account.

Exclusion criteria

- Systematic reviews, case series, commentaries, conference abstracts, letters to editors, technical reports, qualitative studies, and other opinion publications were excluded.
- Studies that were not fully accessible after two emails with the primary/corresponding author were excluded since assessing methodological quality in the absence of the full text was problematic.
- Studies that were not explicitly addressed to RMC, such as those studies conducted on CRC in general outpatient department patients, were not taken into account.
- As potential duplicates, studies conducted in the same area during the same study period were excluded.

Search strategies

The studies had to have been published in English before June 30, 2022. Initially, a comprehensive search of studies was done by using PubMed/MEDLINE, Google Scholar, Science Direct, Scopus, ProQuest, Web of Science, Cochrane Library, and Direct of Open Access Journals. The following keywords were used for the database search: “Respectful”, “Woman-Centered”, “Dignified”, “Friendly”, “Non-Abusive”, “Compassionate”, “Non-discriminatory”, “Maternity”, “Maternal”, “Prenatal”, “Antenatal”, “Delivery”, “Childbirth”, “Postnatal”, “Care”, “ Maternal Health Care”, “Health Service”, “Level”, “Magnitude”, “Prevalence”, “Determinants”, “Associated Factors”, “Predictors”, “Ethiopia”, and “Ethiopian”. To connect those keywords, Boolean operators (AND and OR) and truncation were employed. The following key search terms were used ("Respectful"[All Fields] OR "Woman-Centered"[All Fields] OR "Dignified"[All Fields] OR "Friendly"[All Fields] OR "Non-Abusive"[All Fields] OR "Compassionate"[All Fields] OR "Non-discriminatory"[All Fields]) AND ("Maternity care"[All Fields] OR "Maternal care"[All Fields] OR "Prenatal care"[All Fields] OR "Antenatal care"[All Fields] OR "Delivery service"[All Fields] OR "Childbirth"[All Fields] OR "Postnatal care"[All Fields] OR "postpartum Care"[All Fields] OR "Maternal Health Care"[All Fields] OR "Health Service"[All Fields] OR "Maternity care"[All Fields]) AND "Ethiopia"[All Fields] **(S2File)**. Gray literature searches via Google scholar, Google searching, and Addis Ababa and Jimma University institutional repositories supplemented the electronic database search.

Study selection process

All identified studies were imported into the EndNote XI library and checked for duplication. After removing duplicate articles, three authors (AH, DW, and AT) extracted all articles independently at the title, abstract, and full-text levels. A fourth author (BB) independently reviewed 20% of the removed studies and compiled the screened articles, and any inconsistencies were settled by discussion.

Data extraction

The data were extracted using a Microsoft Excel spreadsheet. Two authors (AH and DW) separately extracted the important data using a pre-setted and piloted data extraction form. The data extraction format comprised the primary author's name, publication year, study year, study design, study area, study setup, sample size, response rate, data collection technique, the proportion of RMC, and adjusted odds ratio(AOR) with their 95% confidence interval.

Risk of bias in individual studies

The methodological quality of included studies was assessed by using Joanna Briggs Institute (JBI) Critical appraisal checklist for prevalence studies[28]. Two reviewers independently rated the quality of the included studies (AH and BB). There are nine parameters in the evaluation tool and each parameter has equal weight. (1) Was the sampling frame appropriate to address the target population? (2) Were study participants sampled appropriately? (3) Was the sample size adequate? (4) Were the study subjects and the setting described in detail? (5) Was the data analysis conducted with sufficient coverage of the identified sample? (6) Were valid methods used for the identification of the condition? (7) Was the condition measured in a standard, reliable way for all participants? (8) Was there appropriate statistical analysis? (9) Was the response rate adequate, and if not, was the low response rate managed appropriately? Each item was assessed as either low or high risk of bias. The evaluators assigned a score of '0' if the study met each specific parameter and a score of '1' if it did not. A composite quality index was computed and the risk of bias was graded as low (0-2), moderate (3 or 4), or high (≥5) **(S3File).** Articles with low and moderate risk of bias were considered for this systematic review and meta-analysis.

Measurement of the outcome of interest

The primary outcome variable of this systematic review and meta-analysis was the level of RMC in Ethiopia, which was determined using the pooled prevalence. The secondary outcome variable was RMC determinants, which were estimated using a pooled AOR with 95 percent CIs. RMC is a universal human right that must be provided to all childbearing women in every health system and is measured by four performance standards (friendly care, timely care,

discrimination-free care, and abuse-free care). When those women received all four performance domains, they were considered to have received RMC[2, 12-14].

Statistical methods and analysis

The data extracted from a Microsoft Excel spreadsheet were exported to the STATA^{TM} 16 statistical software, where all statistical data analyses were undertaken. First, Higgins I-square (I^2) statistics and the Cochran's-Q test were used to determine the presence of statistical heterogeneity across included studies. Heterogeneity was classified as low, moderate, or high when the values of I-square were <25, 50-75, and >75%, respectively[28]. Accordingly, significant heterogeneity was detected [I^2=98.5 %, p-value<0.001]. Thus, a random-effects meta-analysis model with the DerSimonian-Laird method was used to determine the pooled prevalence of RMC. The adjusted Odds Ratios(AOR) from eligible studies were extracted, along with their 95% CIs. The pooled AORs were computed using a random- or fixed-effect model. Finally, forest plots were used to display the pooled estimates for RMC and its determinants, along with their respective 95% confidence intervals.

Publication bias

The presence of publication bias was visually checked by using funnel plots, and a symmetrical, large inverted funnel revealed that the likelihood of publication bias was less likely. Statistical methods such as Egger's and Begg's tests were used to supplement visual assessment, and a p-value <0.05 indicate the likelihood of publication bias.

Additional analyses

Subgroup analyses and heterogeneity

Subgroup analyses were performed based on geographical regions, residence, study year, and publication year. To identify potential sources of heterogeneity across studies, a univariate meta-regression analysis was performed with sample size, publication years, and study years as covariates.

Sensitivity analysis

To assess the influence of a single study on the overall pooled prevalence of RMC, sensitivity analysis was performed using a random-effects model.

Results

Study selection

A total of 1599 studies were found through all searches and 1078 records were duplicates and were thus removed. The remaining 521 studies were eligible for screening. Based on the title and abstract screening, 478 studies were excluded, having left 43 full articles. Twenty-seven studies were removed (twelve owing to insufficient data, seven failed to state the outcome of interest clearly, two case reports, and six were qualitative studies). Finally, 16 studies were considered for systematic review and meta-analysis [29-44] (Fig1).

Fig1. PRISMA flow diagram describing the selection of studies for systematic review and meta-analysis

Characteristics of included studies

Sixteen studies with a total of 6354 study participants were considered [29-44]. All of the eligible studies were cross-sectional in design. The studies were carried out between 2013 and 2021. All of the included studies collected data through face-to-face interviews with a pre-tested, interviewer-administered questionnaire. Studies conducted in Addis Ababa (n=173) [44] and South Nations, Nationalities, and Peoples' Region(SNNPR) (n=783) [37], accounted for the minimum and maximum sample sizes, respectively. In terms of the distribution of the studies across the geographical region, five studies were from Amhara[31, 40-43], five from Oromia[30, 32, 33, 35, 39], two from Addis Ababa[34, 44], two from SNNPR[36, 37], one from Harari[38], and one from Benishangul Gumuz[29]. When it came to the risk of bias in the included studies, the majority (14) had a low risk, with the remaining two having a moderate risk (Table 1).

Table 1: Descriptive summary of studies included in systematic review and meta-analysis of the level of RMC and its determinants in Ethiopia, 2015-2022

Authors name, year of publication	Study year	Region	Study area	Study design	sampling techniques	Sample size	Response rate	RMC	Risk of bias
Amsalu et al., 2022 [29]	2019	Benishangul Gumuz	Benishangul Gumuz	CS	srs	404	97.34	12.65	Low
Yismaw et al., 2022[30]	2019	Oromia	Illu ababora	CS	SRS	281	98.5	47.30	Low
Yalew et al., 2022[31]	2018	Amhara	Dessei	CS	srs	389	99.7	43.40	Low
Eneyew et al., 2021[32]	2021	Oromia	Jimma	CS	srs	348	100	77.00	Moderate
Adane et al., 2021[33]	2019	Oromia	Shashemene	CS	srs	420	99.5	63.00	Low

Ambachew, 2021[34]	2021	Addis Ababa	A.A	CS	srs	384	99.2	65.81	Low
Cafo et al., 2021[35]	2020	Oromia	Wollega	CS	SRS	351	91.4	66.95	Low
Abdo et al., 2021[36]	2020	SNNPR	Hadiya	CS	srs	413	97.86	53.00	Moderate
Wochefu et al, 2021[37]	2019	SNNPR	Hawassa	CS	srs	783	97.11	36.50	Low
Bante et al., 2020 [38]	2017	Harari	Harar	CS	srs	425	100	38.40	Low
Bulto et al., 2020 [39]	2018	Oromia	West Shoa Zone	CS	srs	567	97.5	35.80	Low
Yosef et al., 2020 [40]	2020	Amhara	Northwest Amhara	CS	srs	410	97.16	56.30	Low
Wubetu et al., 2020 [41]	2019	Amhara	Debre Birhan	CS	SRS	412	99.8	35.70	Low
Dagnaw et al.,2020 [42]	2019	Amhara	Dessei town	CS	SRS	310	97.8	64.50	Low
Wassihun and Zeleke, 2018 [43]	2017	Amhara	Bahirdar	CS	srs	284	100	57.0	Low
Asefa and Bekele, 2015 [44]	2013	Addis Ababa	Addis Ababa	CS	SRS	173	100	22.00	Low
Total						6354	98.12		

CS: Cross-sectional study, SRS: systematic random sampling, srs: simple random sampling

The level of RMC in Ethiopia

Because the prevalence estimate varied across studies with significant heterogeneity (I^2= 98.50%; P<0.001), we used a random-effect model with a DerSimonian and Laird method. The overall pooled prevalence of Respectful maternity care in Ethiopia was found to be 48.44% (95% CI: 39.02- 57.87)**(Fig 2)**.

Fig2. Forest plot showing the pooled prevalence estimates of RMC in Ethiopia, 2013-2022

Regarding the main domains of RMC, 78.53 (95% CI: 72.57, 84.48) and 68.95 (95% CI: 64.52, 73.38) of women received discrimination-free and timely care, respectively**.** Only half, 49.99 (95% CI: 32.46, 67.52) of women got friendly care(Table 2).

Table 2: The pooled prevalence of domains of RMC in Ethiopia, 2022

Domains of RMC with a list of studies	Sample size	Pooled prevalence(95% CI)	Heterogeneity		P-value
			I^2	Cochran's Q	
**Friendly care**<br>Amsalu et al., 2022 [29]<br>Yismaw et al., 2022 [30]<br>Adane et al., 2021 [33]<br>Ambachew, 2021 [34]	3175	49.99 (32.46,67.52)	99.2	899.01	0<0.001

Bante et al., 2020[38]<br>Bulto et al., 2020 [39]<br>Yosef et al., 2020[40]<br>Wassihun and Zeleke, 2018[43]					
**Abuse-free care**<br>Amsalu et al., 2022 [29]<br>Yismaw et al., 2022 [30]<br>Adane et al., 2021 [33]<br>Ambachew, 2021 [34]<br>Wochefu et al, 2021[37]<br>Bante et al., 2020[38]<br>Bulto et al., 2020 [39]<br>Yosef et al., 2020[40]<br>Wassihun and Zeleke, 2018 [43]	3958	58.36(46.44,70.29)	98.5	537.34	0<0.001
**Timely care**<br>Amsalu et al., 2022 [29]<br>Yismaw et al., 2022 [30]<br>Adane et al., 2021 [33]<br>Ambachew, 2021 [34]<br>Wochefu et al, 2021[37]<br>Bante et al., 2020[38]<br>Bulto et al., 2020 [39]<br>Yosef et al., 2020[40]<br>Wassihun and Zeleke, 2018 [43]	3958	68.95(64.52, 73.38)	89.4	75.82	0<0.001
**Discrimination- free care**<br>Amsalu et al., 2022 [29]<br>Yismaw et al., 2022 [30]<br>Adane et al., 2021 [33]<br>Ambachew, 2021 [34]<br>Wochefu et al, 2021[37]<br>Bante et al., 2020[38]<br>Bulto et al., 2020 [39]<br>Yosef et al., 2020[40]<br>Wassihun and Zeleke, 2018 [43]	3958	78.53(72.57, 84.48)	96.1	207.03	0<0.001

Subgroup analyses

Subgroup analyses were conducted by region, study year, and publication year. Accordingly, the highest level of RMC was reported in Oromia and Amhara regions, 58.01%(95% CI: 42.44, 73.58), and 51.31%(95% CI: 41.10, 61.53), respectively. On the other hand, the lowest level of RMC was reported in Benishangul Gumuz, 12.65% (95% CI: 9.45, 15.90)**(Fig 3).**

Fig 3. Sub-group analysis for the pooled prevalence of RMC by geographical regions of Ethiopia, 2013-2022.

In addition, We performed a subgroup analysis based on the year when the studies were conducted. Accordingly, the pooled prevalence of RMC was41.15% (95% CI: 28.85–53.44) for studies conducted before 2020 and 68.83% (95% CI: 55.30–72.37) for studies conducted in 2020 and after **(Fig 4)**

Fig 4. Sub-group analysis for the pooled prevalence of RMC by study year in Ethiopia, 2022.

Heterogeneity and publication bias

A univariate meta-regression analysis was run using study-level characteristics (publication year and sample size) as a cofactor to identify the possible source of heterogeneity across the included studies. However, heterogeneity was not explained by sample size (P = 0.582), and the publication year (P= 0.448) (Table 3).

Table 3: A univariate meta-regression analysis of factors affecting between-study heterogeneity

Heterogeneity source	Coefficients	Std.Err	p-value	95% CI
Sample size	-0.0198533	0.036104	0.582	-.0906158, .0509091
Publication year	2.237802	2.948367	0.448	-3.540891, 8.016496

The funnel plot was used to visually examine publication bias, and the effect estimates were asymmetrical, indicating that publication bias was unlikely **(Fig 5)**. Furthermore, we checked the presence of publication bias statistically by running Egger's regression test and an adjusted Beggs rank correlation test and the p values were 0.51 and 0.86, respectively. All of these indicate that the presence of publication bias in this study was unlikely.

Fig 5. Funnel plot displaying publication bias of studies reporting the RMC in Ethiopia, 2022.

Sensitivity analysis

A sensitivity analysis using a random-effects model was carried out to detect the effects of a single study on the overall meta-analysis estimate. As a result, there is no evidence that a single study influenced the pooled prevalence of RMC **(Fig 6).**

Fig 6. Sensitivity analysis for the pooled prevalence of RMC in Ethiopia, 2022

Determinants of RMC

Thirteen variables were extracted from the included studies to identify determinants of RMC(S4File). As significant determinants of RMC, six variables were identified: giving birth during the day, planning status of previous pregnancy, having ANC visit, experiencing an obstetric complication, and receiving service from health care providers who were trained on CRC.

The influence of CRC training on RMC was assessed by using the findings of three studies[29, 30, 39]. Women who received maternal health services from CRC-trained healthcare providers

were 4.09 times more likely than their counterparts to receive RMC. [AOR = 4.09, 95% CI: 1.73, 6.44] **(Fig 7)**.

Fig7. Forest plot showing the association between service delivered by CRC-trained healthcare providers and RMC in Ethiopia, 2022.

Moreover, we used four studies[38-41] to assess the relationship between having ANC and receiving RMC during childbirth. Accordingly, women who received adequate ANC have a 2.34 times greater chance of receiving RMC than their counterparts [AOR = 2.34, 95% CI: 1.62, 3.06]**(Fig 8)**

Fig 8. Forest plot showing the association between ANC and RMC during childbirth in Ethiopia, 2022

The effect of time when the childbirth took place was assessed using the findings of five studies[29, 31, 39-41]. A fixed-effect meta-analysis of AORs revealed that the odds of receiving RMC were 2.61 times higher for women who gave birth during the daytime as compared to those who gave birth at night [AOR: 2.61, 95% CI: 1.92, 3.31]**(Fig 9).**

Fig 9. Forest plot showing the association between daytime delivery and RMC in Ethiopia, 2022.

As per the findings of four studies [30, 33, 38, 39], the pregnancy planning status at the time of childbirth had a positive association with RMC. The likelihood of receiving RMC was 4.43 times higher for those mothers with planned pregnancies as compared to women who came to service delivery points with unplanned pregnancies [AOR: 4.43, 95% CI: 2.74, 6.12]**(Fig 10)**

Fig10. Forest plot showing the association between planned pregnancy and RMC in Ethiopia, 2022

Finally, a negative association was identified between having obstetric complications and RMC. Those women who sustained any obstetric complication had 54% less likely to get RMC than those women who didn't face any obstetric complication [AOR: 0.46, 95% CI: 0.30, 0.61] **(Fig 11).**

Fig 11. Forest plot showing the association between facing obstetric complications and RMC in Ethiopia, 2022

Discussion

Ethiopian HSTP-I and HSTP-II advocate ensuring equitable and timely delivery of quality health care (reliable, patient-centered, and efficient) to all in need[24, 45]. Those plans placed a strong emphasis on providing the best care possible to all people, regardless of age, gender, socioeconomic status, place of residence, or disability status[45]. Safe motherhood must include respect for women's basic human rights, such as autonomy, decency, feelings, preferences, and priorities, in addition to the prevention of illness or death [2, 5]. Currently, WHO recommends providing RMC per the human rights-based approach to reducing maternal and newborn morbidity and mortality by improving women's pregnancy and childbirth experiences and addressing inequities in MNCH care access[6]. Given the importance of RMC in ensuring the quality of MNCH services, assessing its status at the national level allows for a better understanding of its potential contribution to the national and global accomplishment of HSTP[45] and SDG[3, 9], respectively. Hence, this systematic review and meta-analysis was aimed at determining the level of RMC and its determinants in Ethiopia.

The estimated pooled prevalence of RMC in Ethiopia was 48.44% (95% CI: 39.02- 57.87). Accordingly, the level of RMC was higher as compared to findings of a systematic review and meta-analysis in India(28.7%)[46] and a study conducted in East and southern Africa(30%)[47]. In addition, the current finding was higher than primary studies conducted in Pakistan(2.6% and 0.5%)[48, 49], Peru(2.6%)[50], Tanzania(30%)[51] and Nigeria(2%) [52]. This could be due to the Ethiopian government's effort since 2020 to create CRC health care providers as one of the pillars of HSTP-I and -II[24, 45]. Furthermore, there is an increasing commitment and interest in implementing the CRC initiative at the national level through the provision of CRC training to over 27, 000 health leaders and health workers across the country, with a particular emphasis on those who work at MNCH service delivery points[53].

On the other hand, the finding was lower than primary studies conducted in Brazil (81.7%)[54], Mexico (72.3%)[30], India(76%)[55], and Kenya(80.0%)[56]. The disparity in these findings could be attributed to differences in the methodologies and tools used to measure RMC, socio-cultural and economic differences, study period, and organizational factors such as a shortage of health facility to population ratio. Furthermore, it was significantly lower than the Ethiopian government's stated goal of increasing CRC to 90 percent by 2025 in the HSTP-II[45]. Also, the current finding was found to be low as compared to a finding obtained by direct observation of 16 model health facilities in Ethiopia(60.4%)[57]. Hence, the government should work to ensure an adequate number and mix of quality health workforces who are motivated,

competent, and compassionate to enhance RMC. In addition, due consideration should be given to the development of a short-term training manual that improves the awareness and practice of RMC among health workers at service delivery points[45]. The Ministry of Health and Education should take a multi-pronged approach, beginning with the recruitment of students for health science programs and ending with the effective management of health professionals who are already working[58].

As per subgroup analysis results, the highest and the lowest level of RMC was reported in the Oromia, 58.01%(95% CI: 42.44, 73.58) and Benishangul Gumuz region 12.65% (95% CI: 9.45, 15.90) respectively, while it was lowest in. These differences could be attributed to regional disparities in the number of HCPs and health facilities, with the region with the lowest prevalence being one of Ethiopia's emerging regions with the lowest MNCH service coverage[19]. Furthermore, the low prevalence could be attributed to the small number of studies included in this meta-analysis, which included only one study from the Benishangul region. Furthermore, in the last 2-3 years, the Benishangul Gumuz region has been one of the most insecure, with frequent conflicts that have resulted in the displacement of civilians and health care providers due to security concerns[59]. As a result, there may be a lapse in stringently monitoring the MNCH program, resulting in low RMC.

Furthermore, from a sub-group analysis studies conducted since 2020 had the highest level of RMC, 63.83% (95% CI:55.3, 72.37), compared to studies conducted before 2020. The possible justification could be the Ethiopian government's emphasis on addressing a low uptake of maternal health service utilization by improving RMC through various measures, particularly in the previous two years[45]. The measures taken were, creating model professionals in each health facility, advocacy campaigns through mass media, and enacting a Patients' Rights and Responsibilities law[45, 53]. Furthermore, the development and implementation of a generic curriculum in pre-service education, as well as the establishment of well-functioning 16 CRC incubation centers, including national referral and regional hospitals, may have contributed to the good progress of the RMC level over the last three years[58].

Another objective of this systematic review and meta-analysis was to identify the most important factors that affect the level of RMC. Accordingly, receiving service from CRC-trained health care providers, having ANC visits, pregnancy planning status, giving birth during the daytime, and experiencing an obstetric complication were identified as determinants of RMC.

According to the current systematic review and meta-analysis, receiving MNCH services from CRC-trained providers increases the likelihood of receiving RMC. The finding was supported

by studies conducted in India[60, 61], Sanford, USA[62], and Tanzania[63]. It is widely acknowledged that CRC training is vital for gearing up MNHC providers to offer human-centered care, serve patients ethically and with respect, keep taking a professional oath, and promote providers to provide clients with satisfactory service quality[24, 62, 64]. Besides that, the training may influence HCPs' knowledge, motivation, and attitude toward the RMC, which will have a significant positive impact on its provision. As a result, managers in the health sector need to emphasize on the provision of CRC training for health care providers, with a due consideration paid to those who work at maternity service delivery points. The Federal Ministry of Health should collaborate with the Ministry of Education to incorporate CRC issues into the acting curriculum in order to familiarize newly emerging health care providers with the RMC.

In addition, women who received adequate ANC had a greater chance of receiving RMC. This finding was in tandem with studies conducted in Kenya[56] and Tanzania[51]. The possible justification could be that women who had adequate ANC visits had a better chance of acclimating to the health facility setup and developing close relations with the HCP. All of these are essential in ingraining trust in the facility's services, which resulted in RMC[39, 53].

Furthermore, women with planned pregnancies were more likely to receive RMC than those who had not. This could be because women with planned pregnancies are more likely to receive prenatal care services in the same facility where they will give birth, facilitating their interaction with health professionals and ultimately leading to RMC[51]. Furthermore, using MNCH services helps women become acquainted with the service providers, reduces depression, and increases the mother's attitude toward the care as respectful. Evidence indicated that planned pregnancy increases women's contentment and they acknowledge the service provided as reverent[65]. On the other perspective, HCPs should be aware that it is their responsibility to treat all birthing women equally, regardless of their pregnancy planning status. Rather than mistreating women who have had an unplanned pregnancy, it would be recommendable to focus on preventing those very pregnancies through the provision of contraception.

In addition, this meta-analysis discovered that women who gave birth during the day had a higher chance of receiving RMC than those who gave birth at night. The finding was supported by studies conducted in Kenya[66]. This could be because there are more health care providers during the day than at night, when only one health worker may be assigned to duty in health centers. Furthermore, the way senior health workers and managers monitor health care providers during the day may be good, which gives rise to the delivery of RMC. At the national

level, the majority of health facilities had infrastructural problems, such as a lack of electricity[67], and the range of this problem may be lowered during daytime childbirth, enhancing the likelihood of receiving RMC[53]. On the other hand, the tendency to receive low RMC during the night shift may be explained by the low staff number -to- obstetric cases that require nighttime maternity care services (i.e labor starts for most women at night time). Furthermore, health providers may become tired during the night due to workload, and they may not act normally because they are awake from sleep, all of which may result in physical or verbal abuse of the parturient. This may be an implication for human resource managers to assign an adequate number of HCPs to the night-shift duties to reduce workload.

Finally, women who experienced obstetric complications were found to have a lower likelihood of receiving RMC. Studies conducted in India[60] and Tanzania[22] corroborated the current finding. This could be because women who experienced complications during labor are more likely to develop postpartum blues and depression, which can impede and lower the process and level of receiving RMC. In addition, complicated labor necessitates frequent and meticulous follow-up, which exhausts the provider and may result in service abandonment. Furthermore, those women are admitted and stay in health facilities for an extended period with little or no support, and they may perceive the service as unwelcoming, which may result in underreporting of RMC.

This was the first systematic review and meta-analysis of its kind in Ethiopia to assess the level of RMC and its determinants. It could help policymakers and managers at all levels improve the quality of MNCH, which is one of the HSTP and SDG agendas[3, 45]. However, due to some of the limitations listed below, the findings should be interpreted with caution. First, the search only included articles published in English. Because of the nature of the study design, the majority of the studies considered were cross-sectional, making it difficult to establish a cause-effect relationship. Furthermore, the studies were limited to six regions, which may limit the generalizability of the findings. Finally, because of the scarcity of comparable systematic reviews and meta-analyses, we were compelled to discuss some of our findings, particularly the RMC with primary studies conducted outside of Ethiopia.

Conclusion

As per this meta-analysis, the level of RMC in Ethiopia was low (48.44 percent), suggesting that more emphasis is needed to plan and implement intervention measures. Accordingly, receiving service by CRC-trained health care providers, having ANC visits, pregnancy planning status, giving birth during the daytime, and experiencing an obstetric complication were identified as determinants of RMC. Managers in the health sector should give due emphasis to the provision of CRC training for health care providers, who work at maternity service delivery points. Stakeholders in the health sector need to work to increase the uptake of prenatal care to improve client-provider relationships across a continuum of care. Human resource managers should assign an adequate number of HCPs to the night-shift duties to reduce the workload among obstetric providers. A due emphasis should be given to those women who developed an obstetric complication through a continuous follow-up.

Abbreviations

AOR: Adjusted Odds Ratio CRC: Compassionate, Respectful and caring; FMOH: Federal Ministry of Health; HCPs: Health Care Providers; HSTP: Health Sector Transformation Plan; JBI: Joanna Briggs Institute; LMICs: Low and Middle-Income Countries; MNCH: Materna, neonatal and child health; PRISMA: Preferred Reporting Items for Systematic Reviews and Meta-Analysis; RMC: Respectful maternity care; SNNPR: South Nations and Nationalities People of the Region; WHO: World Health Organization.

Funding:

There is no funding to report.

Data Availability

All the data used to strengthen the results of this study are fully available without restriction**.**

Disclosure

We would like to assure you that, with full responsibility and communication, the document has been submitted. We indicated that there is no conflict of interest with other individuals or organizations that could influence or bias the content of the paper inappropriately.

Acknowledgments

We would like to thank Wachemo University, College of Medicine and Health Sciences, for providing us with free internet access while we were conducting this research. We would like to express our gratitude to all of the authors of the studies included in this systematic review and meta-analysis.

Supportive information

S1File: PRISMA checklist 2020 used to report the result of systematic review and meta-analysis

S2File: Examples of the search strategy for systematic review and meta-analysis on the level of RMC and its determinants in Ethiopia, 2022

S3File: JBI Critical Appraisal Checklist for prevalence studies used for assessing the individual quality of all studies included in the systematic review and meta-analysis, 2022

S4File: List of variables considered for estimation of pooled odds ratio

S5File: Minimal data set that is used to estimate the pooled prevalence

References

1. Briscoe, L., T. Lavender, and L. McGowan, *A concept analysis of women's vulnerability during pregnancy, birth and the postnatal period.* Journal of Advanced Nursing, 2016. **72**(10): p. 2330-2345.
2. *WHO. The prevention and elimination of disrespect and abuse during facility-based childbirth WHO statement. World Health Organization [Internet]. 2014;(23). https://apps.who.int/iris/bitstream/handle/10665/134588/WHO_RHR_14.23_eng.pdf*
3. Organization, W.H., *World health statistics 2016: monitoring health for the SDGs sustainable development goals*. 2016: World Health Organization.
4. Koblinsky, M., et al., *Quality maternity care for every woman, everywhere: a call to action.* The Lancet, 2016. **388**(10057): p. 2307-2320.
5. Namusonge, L.N. and J.O. Ngachra, *Respectful Maternity Care Interventions: A Systematic Literature Review.* East African Journal of Health and Science, 2021. **3**(1): p. 45-58.
6. *Oladapo OT, Tunc¸alpO¨ , Bonet M, Lawrie TA, Portela A, Downe S, et al. WHO model of intrapartum care for a positive childbirth experience: transforming care of women and babies for improved health and wellbeing. Bjog. 2018 Jul; 125(8):918. https://doi.org/10.1111/1471-0528.15237 PMID: 29637727.*
7. Hajizadeh, K., et al., *Designing a respectful maternity care guideline: a multiphase study.* Reproductive Health, 2022. **19**(1): p. 1-13.
8. Bohren, M.A., Ö. Tunçalp, and S. Miller, *Transforming intrapartum care: Respectful maternity care.* Best Practice & Research Clinical Obstetrics & Gynaecology, 2020. **67**: p. 113-126.
9. Organization, W.H., *Strategies towards ending preventable maternal mortality (EPMM).* 2015.
10. *WHO Reproductive Health Library. WHO recommendation on respectful maternity care. (February 2018). The WHO Reproductive Health Library. Geneva: World Health Organization.*
11. *World Health Organization. Standards for improving quality of maternal and newborn care in health facilities. Geneva: World Health Organization; 2016.*
12. Reis, V., et al., *Respectful maternity care.* Washington DC: USAID, 2012.
13. Jolivet, R.R., et al., *Operationalizing respectful maternity care at the healthcare provider level: a systematic scoping review.* Reproductive Health, 2021. **18**(1): p. 1-15.
14. *The White Ribbon Alliance for Safe Motherhood. Respectful maternity care: The universal rights of childbearing women. White Ribb Alliance Safe Mother [Internet]; 2011. p. 1–6. Available from: http://whiteribbonalliance.org/wp-content/uploads/2013/10/Final_RMC_Charter.pdf.*
15. Birtwell, B., L. Hammond, and C. Puckering, *'Me and my Bump': An interpretative phenomenological analysis of the experiences of pregnancy for vulnerable women.* Clinical Child Psychology and Psychiatry, 2015. **20**(2): p. 218-238.

16. Belizán, J.M., et al., *Every woman in the world must have respectful care during childbirth: a reflection*. 2020, Springer. p. 1-3.
17. Sadler, M., et al., *Moving beyond disrespect and abuse: addressing the structural dimensions of obstetric violence.* Reprod Health Matt, 2016. **24**.
18. Nair, M., et al., *Facilitators and barriers to quality of care in maternal, newborn and child health: a global situational analysis through meta review.* BMJ Open, 2014. **4**.
19. *CentralStatisticalAgency[Ethiopia]. Ethiopia Mini Demographic and Health Survey 2014 [Internet]. Addis Ababa, Ethiopia; 2014. Available from: https://phe-ethiopia.org/admin/uploads/attachment-1939-Ethiopia_Mini_Demographic_and_Health_Survey_2014-2.pdf.*
20. Jiru, H.D. and E.G. Sendo, *Promoting compassionate and respectful maternity care during facility-based delivery in Ethiopia: perspectives of clients and midwives.* BMJ Open, 2021. **11**(10): p. e051220.
21. Burrowes, S., et al., *Midwives' and patients' perspectives on disrespect and abuse during labor and delivery care in Ethiopia: a qualitative study.* BMC Pregnancy and Childbirth, 2017. **17**(1): p. 1-14.
22. Kruk, M.E., *Disrespectful and abusive treatment during facility delivery in Tanzania: a facility and community survey.* Health Policy Plan, 2018. **33**.
23. Miller, S., et al., *Beyond too little, too late and too much, too soon: a pathway towards evidence-based, respectful maternity care worldwide.* The Lancet, 2016. **388**.
24. *Ethiopian Federal Ministry of Health. Health Sector Transformation Plan 2015/16–2019/20 [Internet]. Addis Ababa, Ethiopia; 2015. 5–29 p. Available from: https://ehia.gov.et/sites/default/files/Resources/HSTP Final Print 2015-11-27 Print size.pdf.*
25. Reis, V., *Respectful maternity care. Country experience*. 2016.
26. Page, M.J., et al., *The PRISMA 2020 statement: an updated guideline for reporting systematic reviews.* Systematic reviews, 2021. **10**(1): p. 1-11.
27. Buccheri, R.K. and C. Sharifi, *Critical appraisal tools and reporting guidelines for evidence-based practice.* Worldviews on Evidence-Based Nursing, 2017. **14**(6): p. 463-472.
28. Institute, J.B., *JBI Checklist for Analytical Cross Sectional Studies. JBI Global, 2020 https://jbi.global/sites/default/files/2019-05/JBI_Critical_Appraisal-Checklist_for_Analytical_Cross_Sectional_Studies2017_0.pdf*.
29. Amsalu, B., et al., *Respectful maternity care among laboring women in public hospitals of Benishangul Gumuz Region, Ethiopia: A mixed cross-sectional study with direct observations.* SAGE Open Medicine, 2022. **10**: p. 20503121221076995.
30. Yismaw, W.S., T.S. Teklu, and H. Panduragman, *Respectful maternity care during facility-based child birth and associated factors in public health facilities of Ilu AbaBor zone, Southwest Ethiopia.* Reproductive Health, 2022. **19**(1): p. 1-7.
31. Yalew, M., et al., *Respectful delivery care and associated factors among mothers delivered in public health facilities of Dessie city, Northeast Ethiopia: a cross-sectional study.* BMC women's health, 2022. **22**(1): p. 1-8.
32. Melkamu, E., G. Tesfaye, and A. Yetwale, *Provision of Respectful Maternity Care During Labor and Delivery and Associated Factors Among Postnatal Mothers at Jimma University Medical Center, Jimma, South West Ethiopia, 2021.* International Journal of Childbirth, 2021. **11**(4): p. 166-177.
33. Adane, D., A. Bante, and B. Wassihun, *Respectful focused antenatal care and associated factors among pregnant women who visit Shashemene town public hospitals, Oromia region, Ethiopia: a cross-sectional study.* BMC women's health, 2021. **21**(1): p. 1-8.
34. Tarekegne, A.A., B.W. Giru, and B. Mekonnen, *Person-Centered Maternity Care During Childbirth and Associated Factors at Selected Public Hospitals in Addis Ababa, Ethiopia, 2021: A Cross-Sectional Study.* 2021.

35. Cafo, J.M., et al., *Compassionate and Respectful Care From Clients' Perspectives in Wollega Zones Hospitals, Wollega, West Ethiopia.* 2021.
36. Abdo, R.A., et al., *Health system responsiveness in maternity care at Hadiya zone public hospitals in Southern Ethiopia: Users' perspectives.* PloS one, 2021. **16**(10): p. e0258092.
37. *Wochefu BB, Abdo AA, Koboto DD (2021) Compassionate and Respectful Maternity Care and Associated Factors among Women Attending Delivery Services at Public Health Facilities of Hawassa City, Southern Ethiopia. Int Arch Nurs Health Care 7:165. doi.org/10.23937/2469-5823/1510165.*
38. Bante, A., et al., *Respectful maternity care and associated factors among women who delivered at Harar hospitals, eastern Ethiopia: a cross-sectional study.* BMC pregnancy and childbirth, 2020. **20**(1): p. 1-9.
39. Bulto, G.A., D.B. Demissie, and A.S. Tulu, *Respectful maternity care during labor and childbirth and associated factors among women who gave birth at health institutions in the West Shewa zone, Oromia region, Central Ethiopia.* BMC Pregnancy and Childbirth, 2020. **20**(1): p. 1-12.
40. Yosef, A., A. Kebede, and N. Worku, *Respectful maternity care and associated factors among women who attended delivery services in referral hospitals in Northwest Amhara, Ethiopia: a cross-sectional study.* Journal of Multidisciplinary Healthcare, 2020. **13**: p. 1965.
41. Wubetu, Y.A., N.T. Sharew, and O.Y. Mohammed, *Respectful Delivery Care and Associated Factors Among Mothers Delivered in Debre Berhan Town Public Health Facilities, Ethiopia.* 2020.
42. Dagnaw, F.T., et al., *Determinants of person-centered maternity care at the selected health facilities of Dessie town, Northeastern, Ethiopia: community-based cross-sectional study.* BMC Pregnancy and Childbirth, 2020. **20**(1): p. 1-10.
43. Wassihun, B. and S. Zeleke, *Compassionate and respectful maternity care during facility based child birth and women's intent to use maternity service in Bahir Dar, Ethiopia.* BMC pregnancy and childbirth, 2018. **18**(1): p. 1-9.
44. Asefa, A. and D. Bekele, *Status of respectful and non-abusive care during facility-based childbirth in a hospital and health centers in Addis Ababa, Ethiopia.* Reproductive health, 2015. **12**(1): p. 1-9.
45. *Ethiopian Federal Ministry of health, Health Sector Transformation Plan II, 2020/21-2024/25, http://repository.iifphc.org/bitstream/handle/123456789/1414/HSTP-II.pdf?sequence=1&isAllowed=y.*
46. Ansari, H. and R. Yeravdekar, *Respectful maternity care during childbirth in India: A systematic review and meta-analysis.* Journal of Postgraduate Medicine, 2020. **66**(3): p. 133.
47. Rosen, H.E., et al., *Direct observation of respectful maternity care in five countries: a cross-sectional study of health facilities in East and Southern Africa.* BMC pregnancy and childbirth, 2015. **15**(1): p. 1-11.
48. Azhar, Z., O. Oyebode, and H. Masud, *Disrespect and abuse during childbirth in district Gujrat, Pakistan: a quest for respectful maternity care.* PloS ONE, 2018. **13**.
49. Hameed, W. and B.I. Avan, *Women's experiences of mistreatment during childbirth: a comparative view of home-and facility-based births in Pakistan.* PloS One, 2018. **13**.
50. Montesinos-Segura, R., et al., *Disrespect and abuse during childbirth in fourteen hospitals in nine cities of Peru.* International Journal of Gynecology & Obstetrics, 2018. **140**(2): p. 184-190.
51. Sando, D., et al., *The prevalence of disrespect and abuse during facility-based childbirth in urban Tanzania.* BMC Pregnancy Childb, 2016. **16**.
52. Okafor, I.I., E.O. Ugwu, and S.N. Obi, *Disrespect and abuse during facility-based childbirth in a low-income country.* Int J Gynecol Obstet, 2015. **128**.

53. Nigussie, A., et al., *The role of Performance appraisal system on Compassionate, Respectful, and Caring health care delivery in Northwest Ethiopia: an implementation study.* Ethiopian Journal of Health and Biomedical Sciences, 2022. **12**(1SpecialIssue): p. 95-110.
54. Mesenburg, M.A., et al., *Disrespect and abuse of women during the process of childbirth in the 2015 Pelotas birth cohort.* Reprod Health, 2018. **15**.
55. Bhattacharya, S. and T.K. Sundari Ravindran, *Silent voices: institutional disrespect and abuse during delivery among women of Varanasi district, northern India.* BMC Pregnancy Childbirth., 2018. **18**.
56. Abuya, T., *Exploring the prevalence of disrespect and abuse during childbirth in Kenya.* PloS One, 2015. **10**.
57. Jemal, K., et al., *Evaluation of compassionate and respectful care implementation status in model healthcare facilities: a cross-sectional study.* Archives of Public Health, 2022. **80**(1): p. 84.
58. Nigusie, A., et al., *Status of Compassionate, Respectful, and Caring Health Service Delivery: Scoping Review.* JMIR human factors, 2022. **9**(1): p. e30804.
59. Fessha, Y.T. and B. Dessalegn, *Federalism in Ethiopia: Emergence, Progress and Challenges.* Emerging Federal Structures in the Post-Cold War Era, 2022: p. 215-231.
60. Dey, A., et al., *Discordance in self-report and observation data on mistreatment of women by providers during childbirth in Uttar Pradesh, India.* Reprod Health, 2017. **14**.
61. Chaturvedi, S., S. Upadhyay, and A. De Costa, *Competence of birth attendants at providing emergency obstetric care under India's JSY conditional cash transfer program for institutional delivery: an assessment using case vignettes in Madhya Pradesh province.* BMC pregnancy and childbirth, 2014. **14**(1): p. 1-11.
62. Chapin, H.L., et al., *Pilot study of a compassion meditation intervention in chronic pain.* Journal of compassionate health care, 2014. **1**(1): p. 1-12.
63. Webber, G., B. Chirangi, and N. Magatti, *Promoting respectful maternity care in rural Tanzania: nurses' experiences of the "Health Workers for Change" program.* BMC Health Services Research, 2018. **18**(1): p. 1-6.
64. Shea, S. and C. Lionis, *Introducing the journal of compassionate health care*. 2014, Springer. p. 1-2.
65. Bazant, E.S. and M.A. Koenig, *Women's satisfaction with delivery care in Nairobi's informal settlements.* International Journal for Quality in Health Care, 2009. **21**(2): p. 79-86.
66. Abuya, T., et al., *The effect of a multi-component intervention on disrespect and abuse during childbirth in Kenya.* BMC Pregnancy Childbirth, 2015.
67. Lagebo, B.W. and S.M. Mekonnen, *Challenges and approaches to scaling and standardizing health information infrastructure in developing countries: Case studies from Ethiopia*. 2005.

Figure Click here to access/download;Figure;Fig1.tif

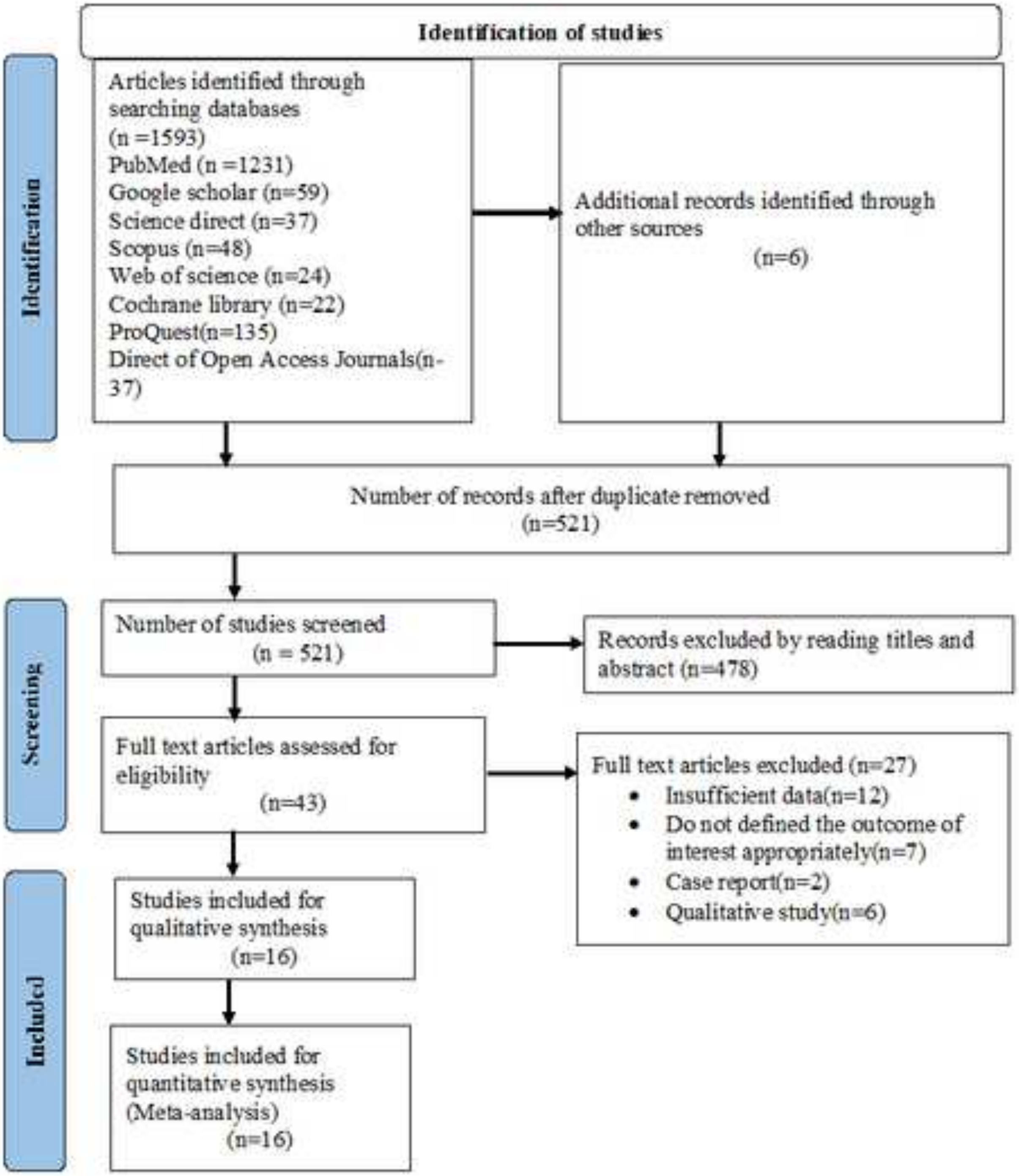

Figure

Click here to access/download;Figure;Fig2.tif

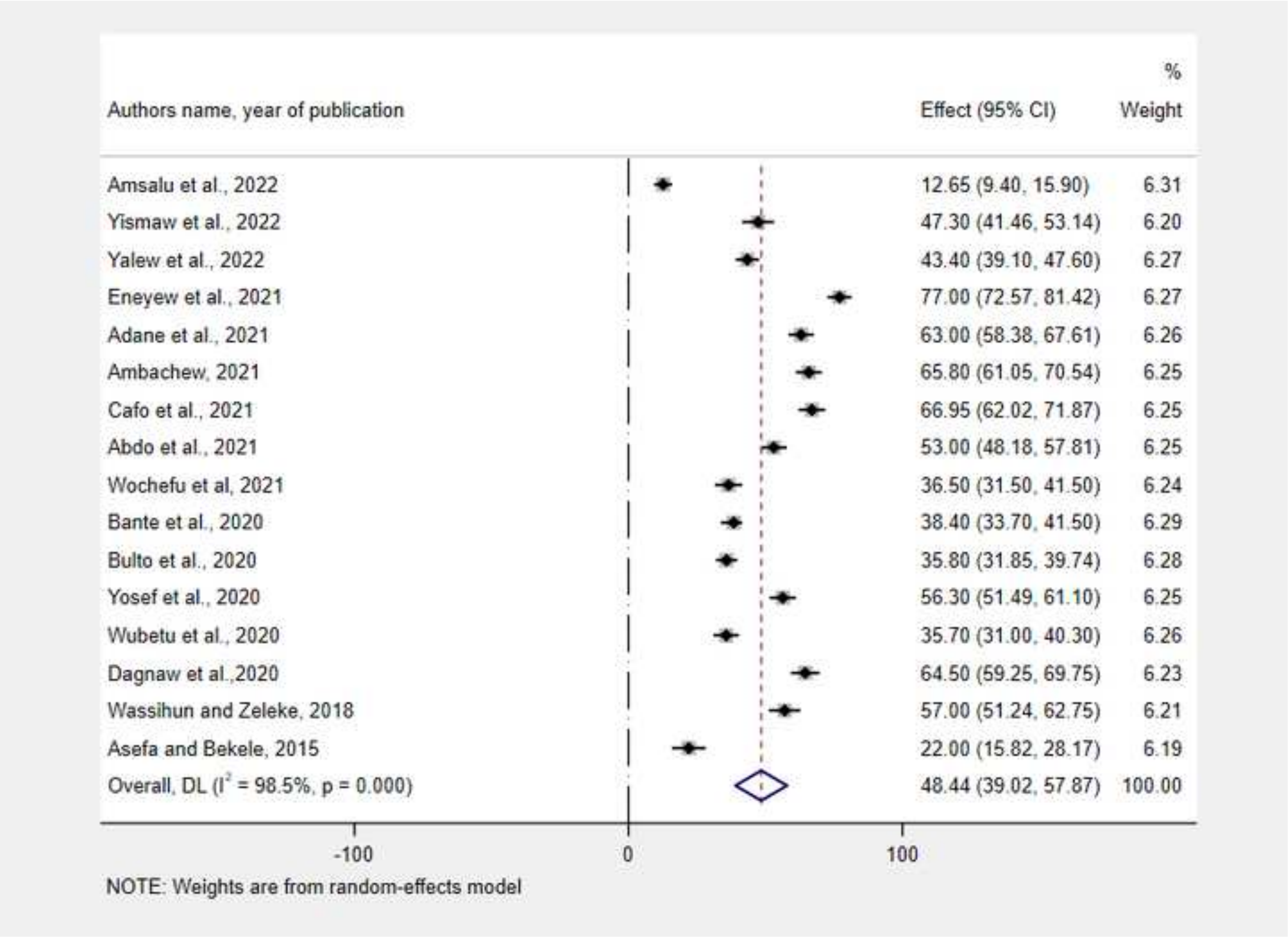

Figure

Click here to access/download;Figure;Fig3.tif

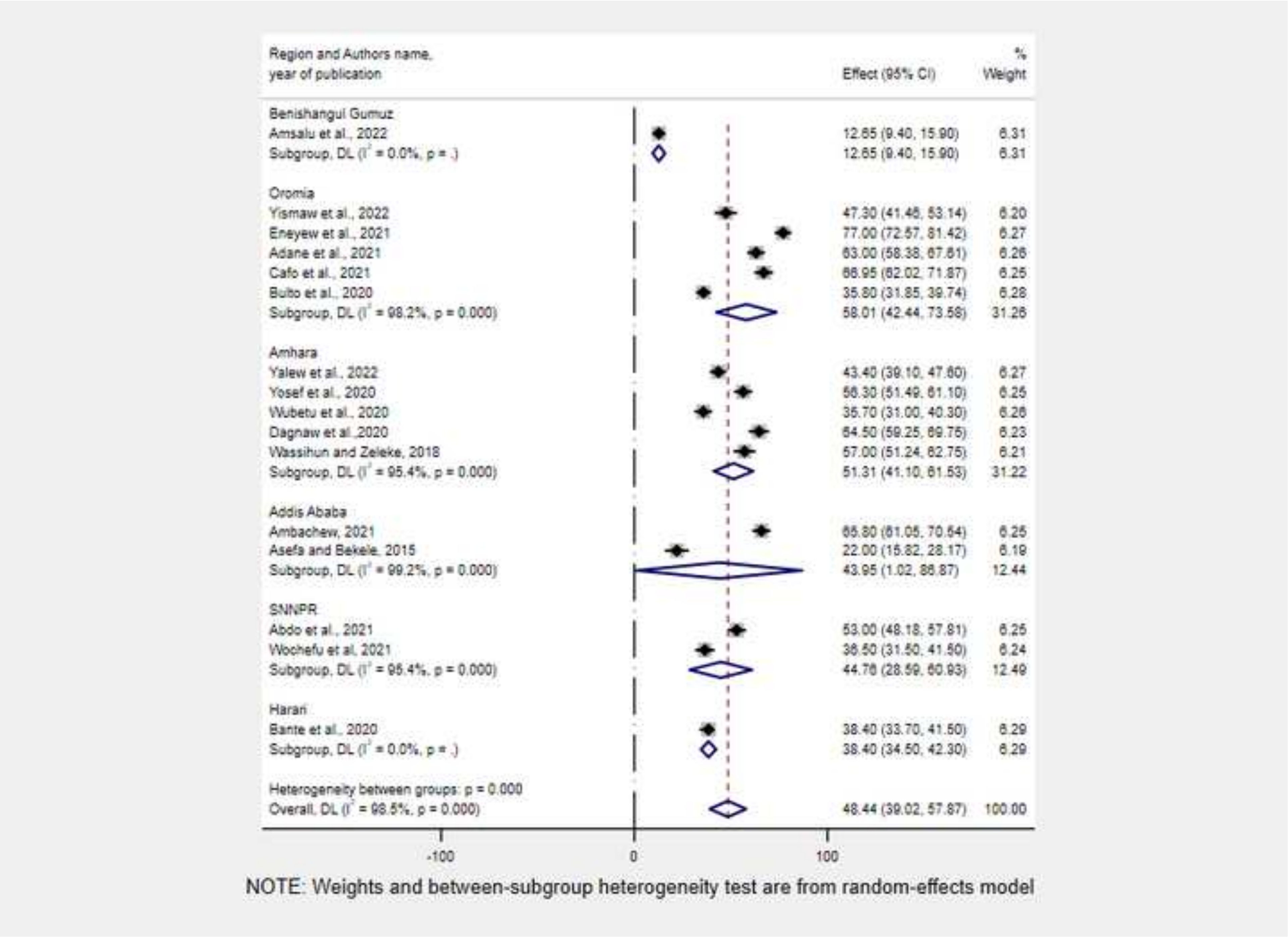

Figure

Click here to access/download;Figure;Fig4.tif

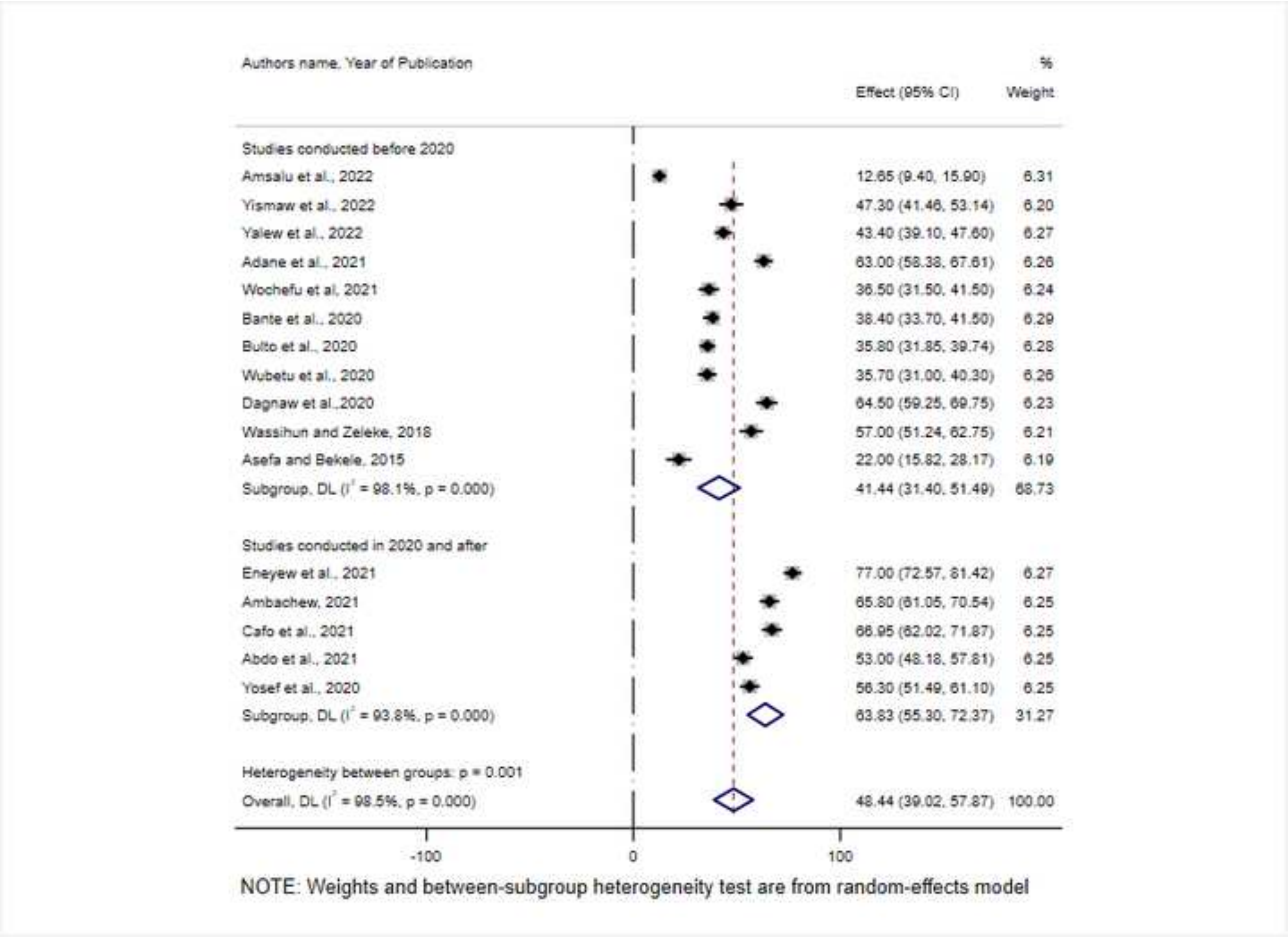

Click here to access/download;Figure;Fig5.tif

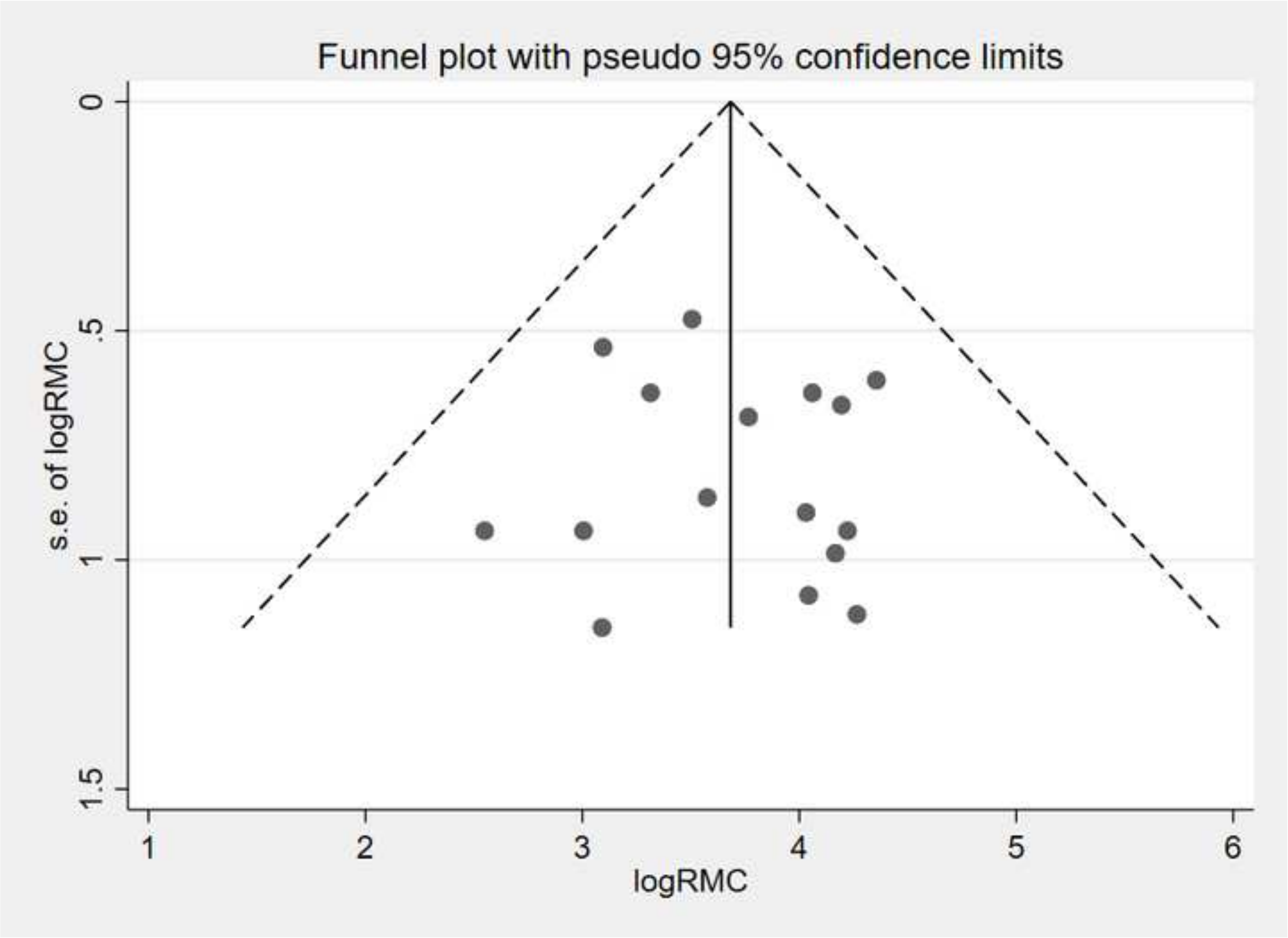

Figure

Click here to access/download;Figure;Fig6.tif

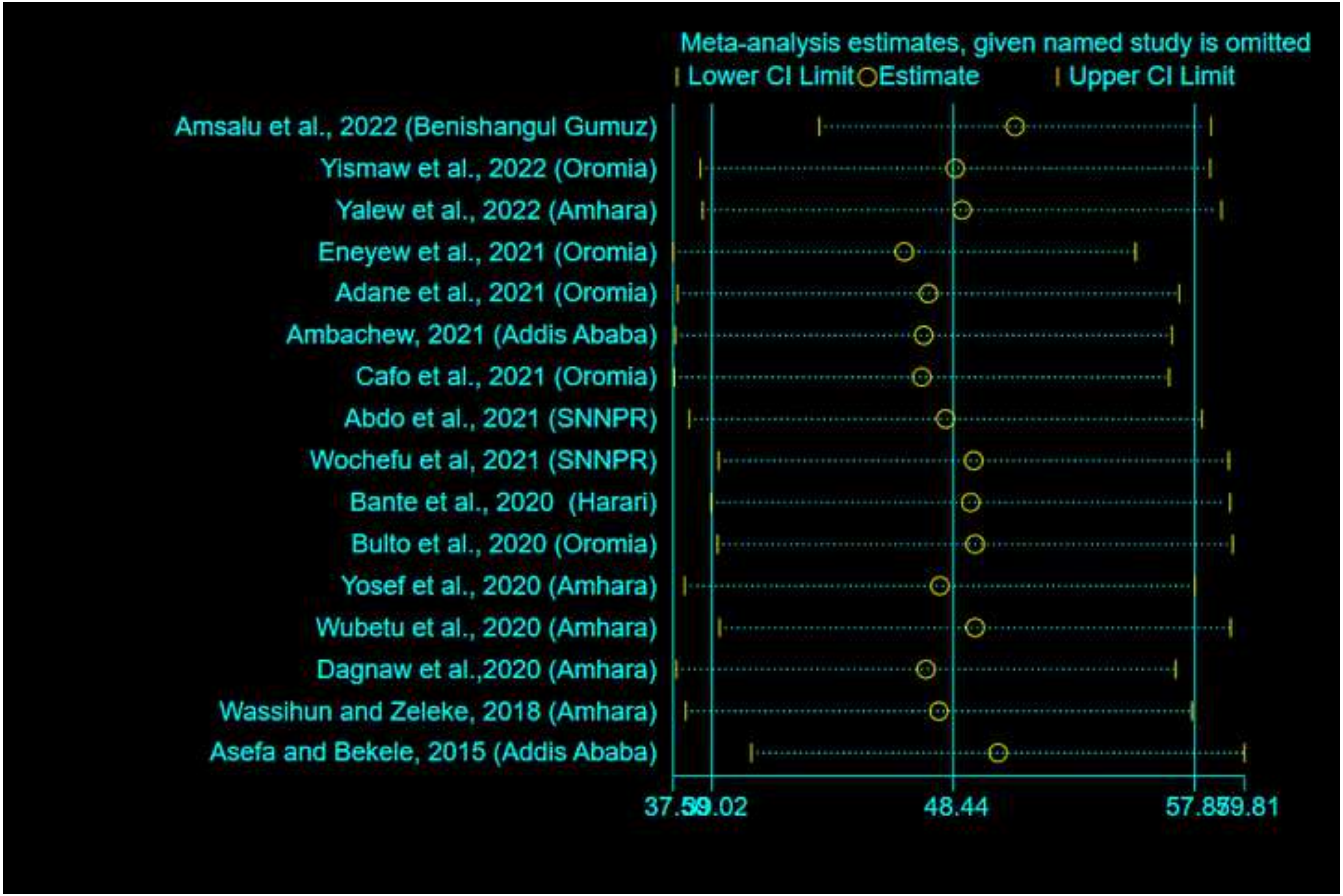

Figure

Click here to access/download;Figure;Fig7.tif

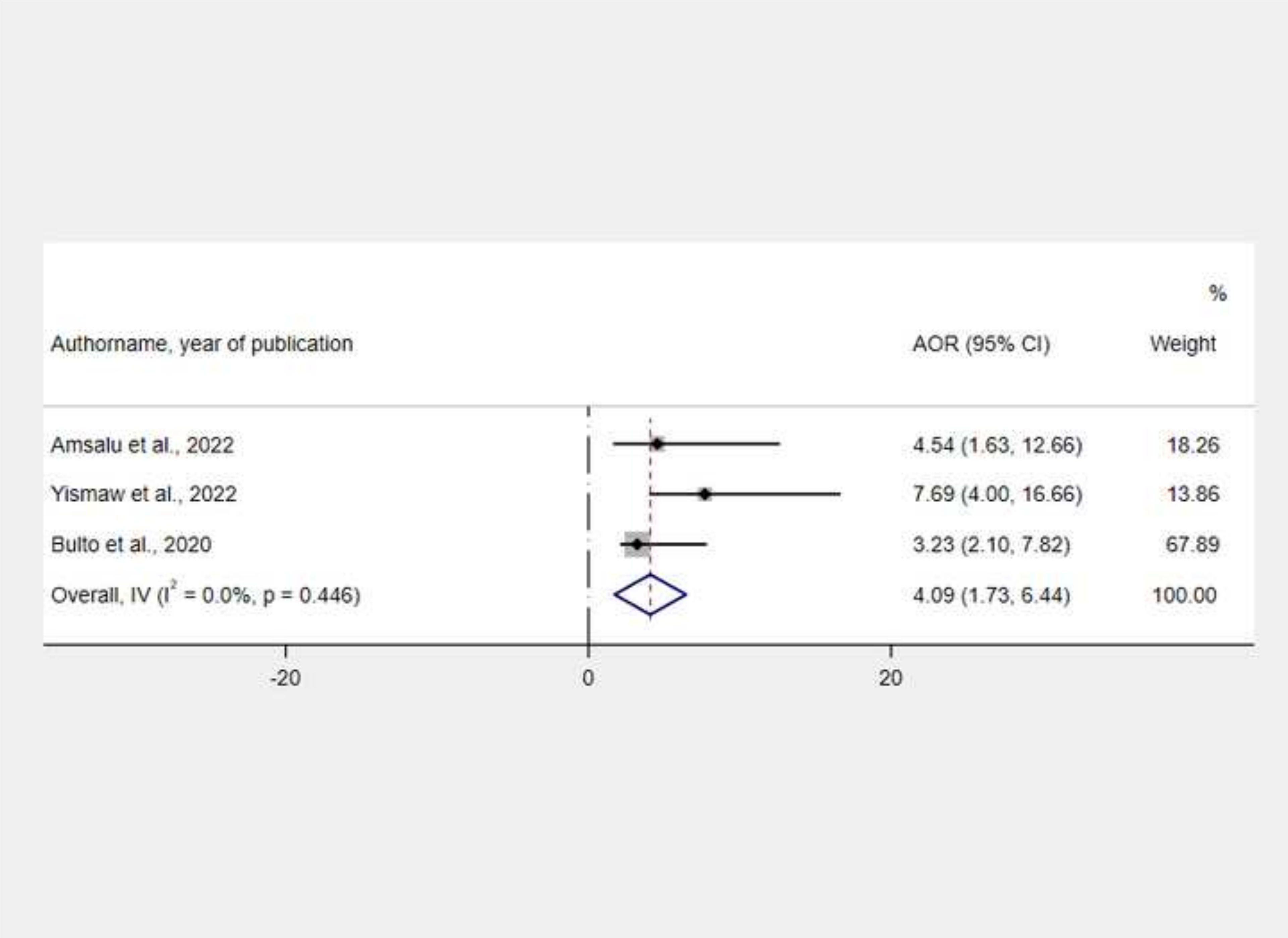

Click here to access/download;Figure;Fig8.tif

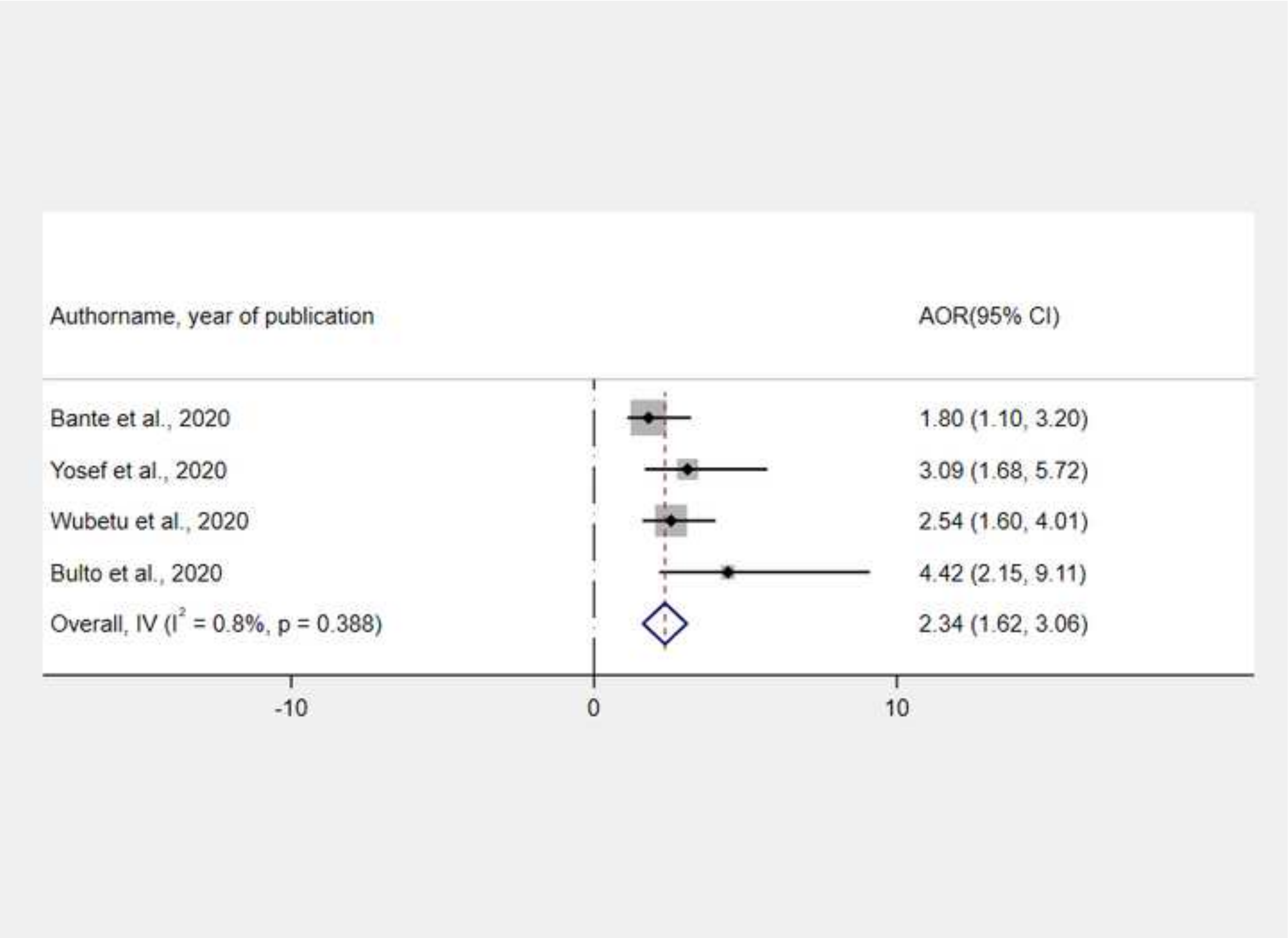

Click here to access/download;Figure;Fig9.tif

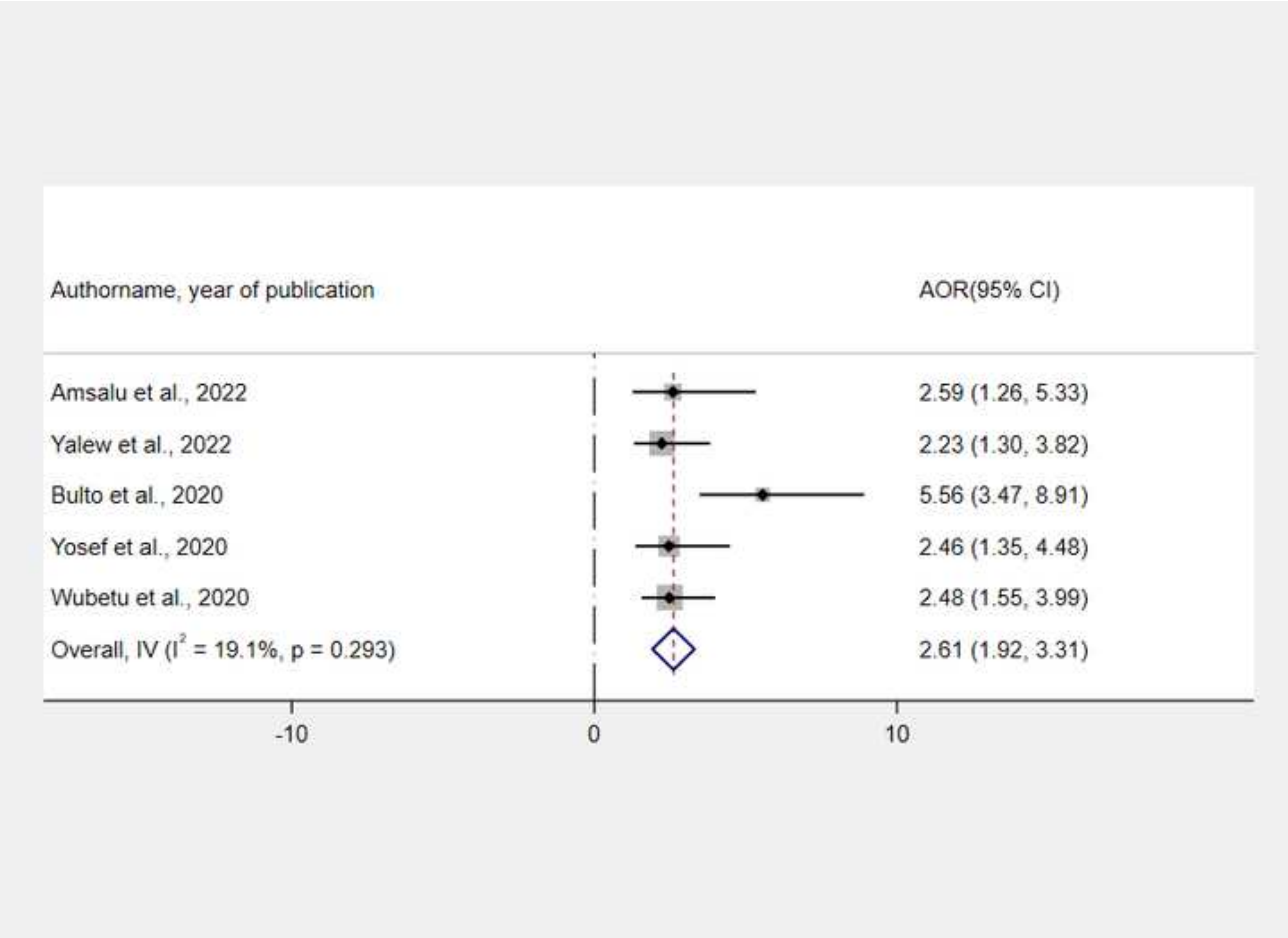

Figure

Click here to access/download;Figure;Fig10.tif

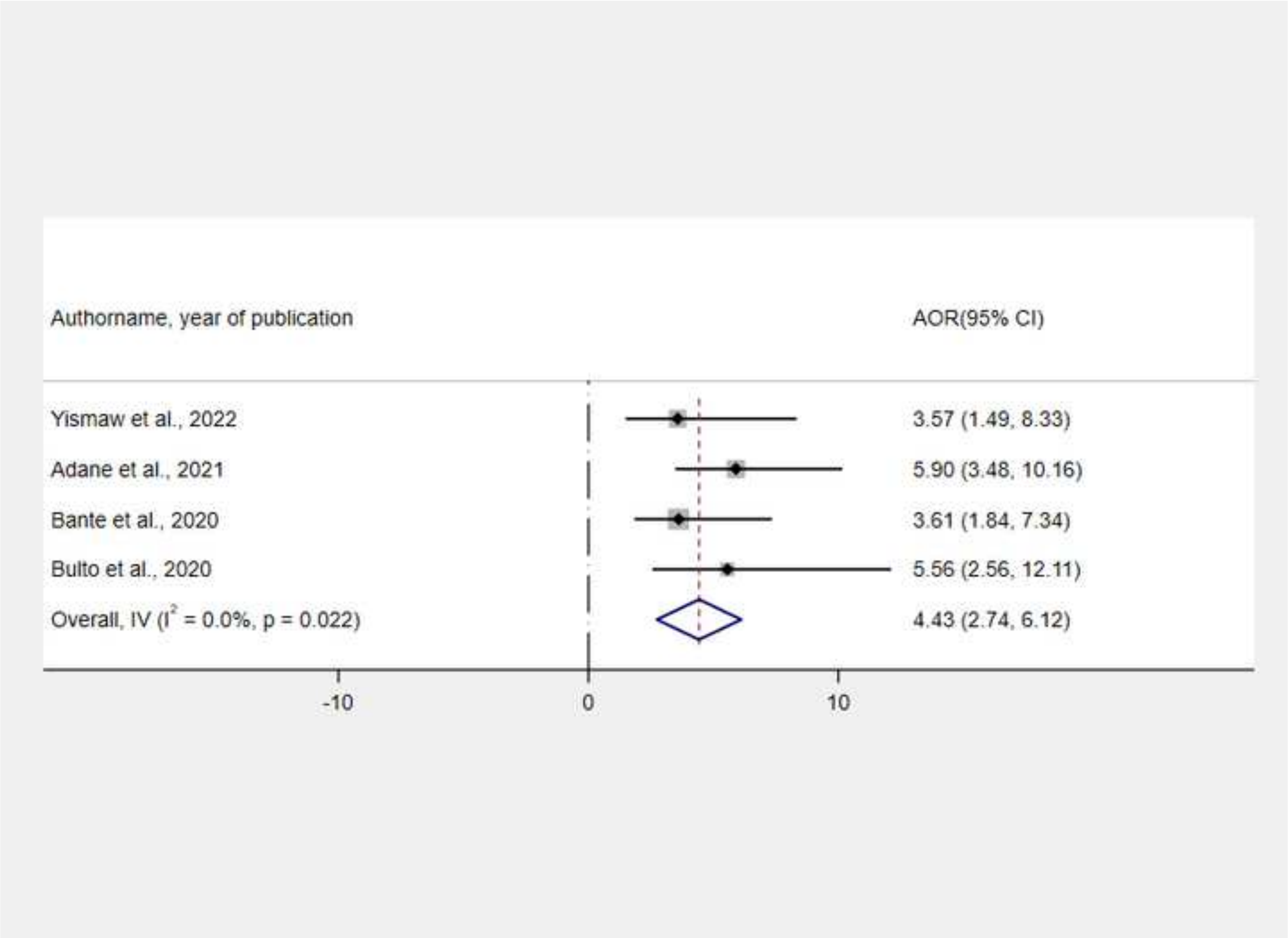

Figure   Click here to access/download;Figure;Fig11.tif

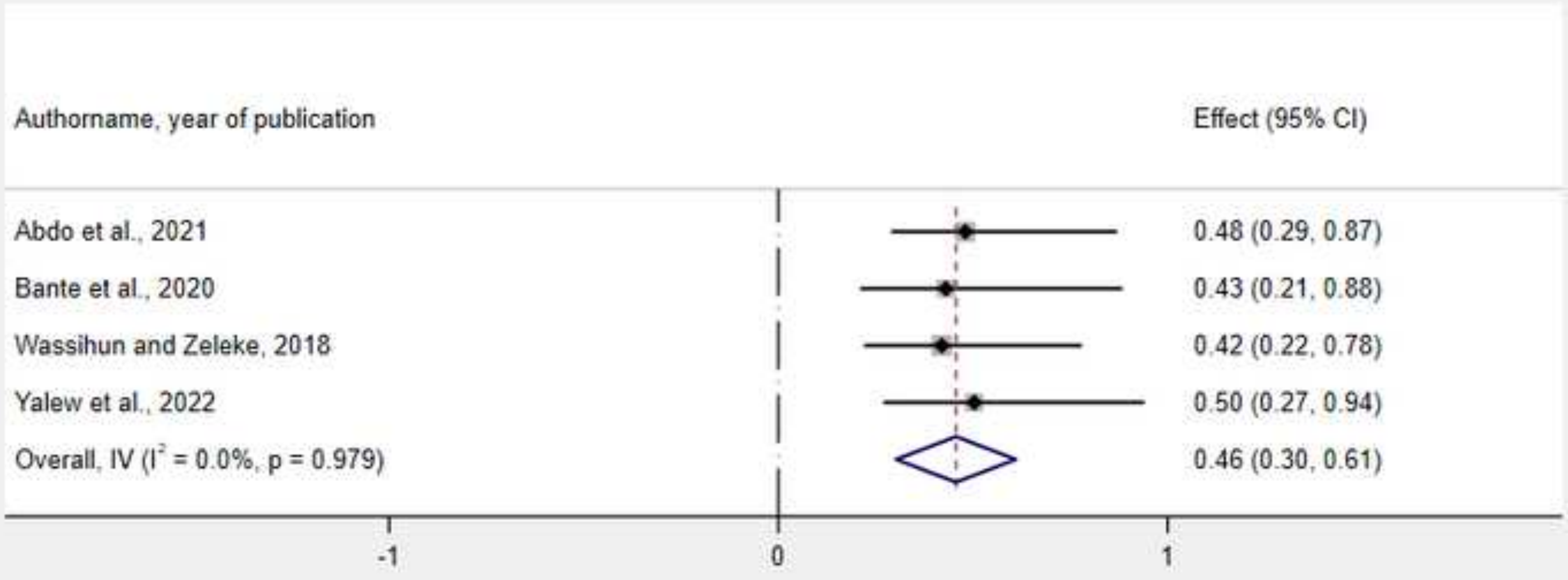

Click here to access/download
Supporting Information
S1File.docx

Click here to access/download
Supporting Information
S2File.docx

Click here to access/download
Supporting Information
S3File.docx

Click here to access/download
Supporting Information
S4File.xlsx

Click here to access/download
Supporting Information
S5File.dta
