# Supplementary material for: The prevalence of respectful maternity care during childbirth and its determinants in Ethiopia: A systematic review and meta-analysis
Source: PLoS One. 2022 Nov 23;17(11):e0277889. doi: 10.1371/journal.pone.0277889 (PMC9683616; doi:10.1371/journal.pone.0277889)
Supplement: Attachment — Submitted filename: Response to Reviewers.docx [file pone.0277889.s007.docx]

**AUTHORS’ RESPONSE TO ACADEMIC EDITOR AND REVIEWERS**

1. **Author’s Response To Academic Editor**

**Dear: Zemenu Yohannes Kassa, Msc (Academic Editor)**

We thank you for a thorough reading and constructive comments and suggestions on our manuscript and for the opportunity to revise and resubmit. We are pleased to submit the revised research article “**The prevalence of respectful maternity care during childbirth and its determinants in Ethiopia: A contemporaneous systematic review and Meta-analysis**” for your consideration in the special collection of Plos One. On the following pages, you will find our responses to the questions raised from your side and two reviewers' comments. On behalf of my co-authors, I thank you for your consideration of this resubmission. We appreciate your time and look forward to your response.

Sincerely,

Aklilu Habte(MPH)(corresponding author)

[**aklilihabte57@gmail.com**](mailto:aklilihabte57@gmail.com)

**Dear Dr. Habte,**

Thank you for submitting your manuscript to PLOS ONE. After careful consideration, we feel that it has merit but does not fully meet PLOS ONE’s publication criteria as it currently stands. Therefore, we invite you to submit a revised version of the manuscript that addresses the points raised during the review process.

**General comment and suggestion**

The topic of the manuscript is interesting. Nevertheless, the reviewers raised several concerns: considering this point, I invite authors to perform the required major revisions.

\# You should modify the title “Determinants of respectful maternity care during childbirth in Ethiopia: A systematic review and Meta-analysis”

You should give line numbers across the manuscript

**Response:** Thank you for providing us with the opportunity to revise our work by incorporating all of the valuable comments and suggestions made by the reviewers. We are also grateful for the reviewers' insightful comments and suggestions, which we received all of them were beneficial for further improving the manuscript.

We have amended the title as per your suggestion and highlighted it on the “title page” of the “Revised manuscript with track changes” Line 1-3, Page1. We also the line number for the revised version of the manuscript

**Comments in the background section**

**Comment 1:** The background is too long, make shortened. The first and second sentences, it is difficult to understand. Write clearly and understandable way.

**Response**: thank you for your comment. As per your suggestion, we have tried to shorten the background section of the abstract by clarifying the vague statements. The corrected version was highlighted in the “Abstract” section of “Revised manuscript with track changes” Line 33-34, Page2.

**Comment 2:** Methods from when to June 2022?

**Response:** It was from 2013 and we have highlighted it in the “Abstract” section of “Revised manuscript with track changes” Line 41, Page2.

Comment3: In the abstract abbreviation does not recommend (CRC and HCPs).

**Response:** We have removed all abbreviations and written them in their full length.

**Comment4:** As per this meta-analysis, the level of RMC in Ethiopia was low (48.44 percent), suggesting that more emphasis is needed to plan and implement intervention measures. This sentence needs modification and avoids the word level.

**Response:** Thank you for your suggestion and we have shortened the statement accordingly. the revised statement is highlighted in the “Abstract” section of the “Revised manuscript with track changes” Line 60, Page2.

**Comment 5:** You should forward your recommendation based on your pertinent findings.

**Response:** thank you for the comment. We have tried to make all our recommendations based on the findings of the study.

**Comment6**: The introduction is too long, you should be focused and addressed your objectives, what is known and what is not unknown. This article is similar to your manuscript <https://pubmed.ncbi.nlm.nih.gov/30760318/>.

**Response:** Thank you for the inquiry, this salient point has been noted and welcomed by

the author team. Significant revisions have thus been made throughout the “introduction” section of the revised manuscript by removing some unnecessary and redundant statements. There were some differences between the above-mentioned article by Mr. Zemenu Yohannes Kassa and the current studies. First, the current SRMA contains more studies(2013-2022) than the previous one(studies done since 2018), which may give in a more comprehensive and representative figure of RMC ta the national level. Furthermore, most of the studies included in the previous SRMA focused solely on disrespect and abuse, unlike the current one.

**Comment 7:** Population: Women in the reproductive age group (15-49), Your population is childbirth, not reproductive age

**Response:** we agree with your suggestion and we have corrected and highlighted it in the “Eligibility criteria” section of the “Revised manuscript with track changes” Line 140, Page5.

**Comment 8:** Study settings are either facility based on community-based

**Response:** thank you for your valuable suggestion. We corrected and highlighted it in the “Eligibility criteria” section of the “Revised manuscript with track changes” Line 145, Page5.

**Comment 9:** Why do you exclude qualitative studies? Why not synthesize evidence from these studies?

**Response:** Thank you for your valuable and pertinent question, which we acknowledge as a limitation of the current study because we failed to consider qualitative studies. We merely focus on the quantitative findings to estimate the pooled prevalence. Your comment shows us the way to focus on qualitative studies and we will do it in the future.

**Comment 10:** the discussion is too long. You should discuss your pertinent findings, how and why this result comes, and the limitations.

**Response:** thank you for your suggestion. We tried to shorten the discussion as per your suggestion by removing some lengthy and redundant statements.

**Journal Requirements:**

When submitting your revision, we need you to address these additional requirements.

**Comment1:** Please ensure that your manuscript meets PLOS ONE's style requirements, including those for file naming. The PLOS ONE style templates can be found at

<https://journals.plos.org/plosone/s/file?id=wjVg/PLOSOne_formatting_sample_main_body.pdf> and

<https://journals.plos.org/plosone/s/file?id=ba62/PLOSOne_formatting_sample_title_authors_affiliations.pdf>

**Response:** Thank you for your suggestion. We have submitted the manuscript as per the journal's requirements. Symbols for each author's contributions were added accordingly and highlighted in the “title page” of the “Revised manuscript with track changes” Line 4-5, Page1.

**Comment2:** Please upload a new copy of Figures 3 and 4 as the detail is not clear. Please follow the link for more information: https://blogs.plos.org/plos/2019/06/looking-good-tips-for-creating-your-plos-figures-graphics/" <https://blogs.plos.org/plos/2019/06/looking-good-tips-for-creating-your-plos-figures-graphics/>

**Response**: thank you for your comment we corrected them according to the journal’s requirement.

Comment3: We note that this manuscript is a systematic review or meta-analysis; our author guidelines, therefore, require that you use PRISMA guidance to help improve reporting quality of this type of study. Please upload copies of the completed PRISMA checklist as Supporting Information with the file name “PRISMA checklist”.

**Response:** We already added it during the initial submission of the manuscript in the “Supportive information” section as “S1File”. We kindly suggest you see Line512-513, Page18 of the “Revised manuscript with track changes”

_____________________________END________________________________________

THANK YOU!!!

**AUTHORS’ RESPONSE TO REVIEWERS**

**Dear: Reviewers**

We thank you for a thorough reading and constructive comments and suggestions on our manuscript and for the opportunity to revise and resubmit. We are pleased to submit the improved research article **“The prevalence of respectful maternity care during childbirth and its determinants in Ethiopia: A contemporaneous systematic review and Meta-analysis”** for your consideration in the special collection of Plos One. On the following pages, you will find our response to the reviewers’ comments. On behalf of my co-authors, I thank you for your consideration of this resubmission. We appreciate your time and look forward to our responses to the comments and suggestions raised from your side independently as “Authors’ response to Reviewers 1, 2,3, and 4”

Sincerely,

Aklilu H. (MPH)(corresponding author)

[**aklilihabte57@gmail.com**](mailto:aklilihabte57@gmail.com)

**Authors’ Response to Reviewer**#**1**

**General comments**

This is an important and methodologically sound article, which will be of great interest and practical utility to a local audience as well as a global one. Nevertheless, some minor revisions are necessary to clarify significant ambiguities stemming mostly from language issues, and the paper would be strengthened overall by good copyediting to improve the quality of the writing and thus the clarity of the content.

**Response:** Thank you very much for taking the time to review our work and for your positive feedback. We got all your constructive comments and suggestions as valuable input for the readability of our work and we have tried to respond as follows. We give due emphasis to those grammatical and Punctuation errors by involving experts in language and literature and we have tried to correct them in the revised version of the manuscript.

**Specific comments**

**Comment1:** In the abstract (but not the body of the paper) there is a typographical error: “DerSimonian Laired”.

**Response:** Thank you for your comment. We have corrected and highlighted in the “Abstract” section of the “Revised manuscript with track changes” Line 48, Page2.

**Comment 2:** CRC: this abbreviation appears throughout the paper, in reference to some aspect of the Ethiopian government’s Health Sector Transformation Plan that emphasizes “compassionate, respectful care”. It is unclear if this is a specific training program with a defined curriculum, learning objectives and outcome measures, such that it could be replicated with similar results. These details are important as it emerges as a significant variable in the logistical regression reported. If so, the program should be described and cited; from a language perspective, it should appear with first letters capitalized. If not, I would suggest citing the HSTP, and adding some discussion and calls for future research to identify what elements of that program are effective in strengthening RMC.

**Response:** thank you for your insightful remarks. After the federal ministry of health launched CRC as one of the pillars of a health sector transformation plan many in-service pieces of training were provided to healthcare providers with a due emphasis on those healthcare providers who work on maternal health service delivery points. So, those studies included in the current SRMA also define training CRC from that perspective. We have already cited the document of HSTP in reference number 20. We have corrected the issue raised from the language perspective in the “Introduction” section of the “Revised manuscript with track changes” Line 107, Page4.

**Comment3**: What is Reference 3 and how is it relevant to the point? There are two rights-based frameworks, White Ribbon and Khosla et al. that I suggest should be cited here.

**Response:** Thank you for your suggestion and now we made changes in the references as per your valuable suggestion and highlighted in the “Introduction” section of the “Revised manuscript with track changes” Line 70, Page 2.

**Comment 4:** I am not aware of studies that expressly measure the prevalence of RMC; many measures the prevalence of Disrespect and Abuse (D&A)/mistreatment. Does this study derive the prevalence of RMC from studies that aim to measure D&A, and if so by what methodology? Or are these all studies that specifically measure RMC, and if so, how was RMC defined and operationalized in these original studies? Was it the same?

**Response:** We appreciate your valuable comments and suggestion. Regarding the measurement of the outcome of interest, we duly emphasize those studies conducted on respectful maternity care/ woman-centered care/ person-centered care/ compassionate and respectful care. Of course, the reveres of D&A might be RMC, but some of the tools used by the researchers to assess D&A were not consistent as compared to the tools used to measure RMC. We kindly request you to assure the included studies were merely dealing with RMC.

**Comment5:** Similar comment to the above: There are so few studies that measure RMC and there have been no standard definitions for RMC to my knowledge other than this paper (https://pubmed.ncbi.nlm.nih.gov/34598705/) that I am concerned whether the studies truly measured prevalence of RMC or whether they looked rather at D&A/mistreatment and extrapolated RMC. RMC cannot be construed as the simple absence of D&A, although this becomes nuanced. It would be important to understand how the authors calculated the prevalence of RMC in the base studies.

**Response:** We appreciate your valuable comments and suggestion. Regarding the measurement of the outcome of interest, we duly emphasize those studies conducted on respectful maternity care/ woman-centered care/ person-centered care/ compassionate and respectful care. Of course, the reveres of D&A might be RMC, but some of the tools used by the researchers to assess D&A were not consistent as compared to the tools used to measure it. We have raised the way of measurement of the outcome variable(RMC) in the “Measurement of the outcome of interest” section of the “Revised manuscript with track changes” Line 214-127, Page7.

**Comment 6:** the following sentence, “RMC is a universal human right that must be provided to all childbearing women in every health system and is measured by four performance standards (friendly care, timely care, discrimination-free care, and abuse-free care),” What is the citation for this? Or was this the study definition? If so, it should be explicitly stated and operational definitions provided.

**Response:** of course, it is the definition and we have already incorporated it in the “Measurement of the outcome of interest” section of the “Revised manuscript with track changes” Line 213-216, Page7.

**Comment 7:** The sentence, “All of the included studies collected data through face-to-face interviews with a pre-tested, interviewer-administered questionnaire,” in what setting? In what timeframe relative to birth?

**Response:** Almost all (15) of the 16 studies were facility-based cross-sectional studies with a maximum time gap of 6 months between childbirth and study. To accommodate some disparities that might be raised due to the study setting and time frame, we used a random-effect model with a DerSimonian and Laird method.

**Comment8:** the following sentence, “As significant determinants of RMC, six variables were identified: giving birth during the day, planning status of previous pregnancy, having ANC visit, experiencing an obstetric complication, and receiving service from health care providers who were trained on CRC.

**Comment8.1:** Does this refer to the current pregnancy, or a previous pregnancy resulting in previous birth? I think it means the current pregnancy preceding the current birth. Please clarify.

**Response**: it was to mean the current pregnancy and we corrected and highlighted it in the “Determinants of RMC” section of the “Revised manuscript with track changes” Line 313, Page 12.

**Comment 8.2:** Does this refer to any ANC or adequate ANC? I believe from reading the whole paper refers to adequate ANC, but this should be clarified. These are significantly different.

**Response:** It was corrected as adequate ANC and highlighted in the “Determinants of RMC” section of the “Revised manuscript with track changes” Line 314, Page 12.

**Comment 8.3.** Most importantly, the directionality of the association between obstetric complications and RMC is NEGATIVE. This MUST be clarified. The way that these four significant factors are lumped together and jointly described as determinants of RMC is very misleading and confusing. The study results demonstrate that obstetric complications are a determinant of reduced odds of RMC, the opposite of the three other variables. This distinction should be made very clear every time it is mentioned.

**Response:** As supported by various studies obstetric complication was negatively associated with RMC. The possible justifications could be because women who experienced complications during labor are more likely to develop postpartum blues and depression, which can impede and lower the process and level of receiving RMC. In addition, complicated labor necessitates frequent and meticulous follow-up, which exhausts the provider and may result in service abandonment. Furthermore, those women are admitted and stay in health facilities for an extended period with little or no support, and they may perceive the service as unwelcoming, which may result in underreporting of RMC. We have tried to mention those possible justifications in the “Discussion” section of the “Revised manuscript with track changes” Line 458-464, Page 16.

**Comment 9**: Page 15: Re. this sentence, “As per subgroup analysis results, the highest and the lowest level of RMC was reported in the Oromia, 58.01%(95% CI: 42.44, 73.58) and Benishangul Gumuz region 12.65% (95% CI: 9.45, 15.90) respectively, while it was lowest in.” Correct significant typos and missing words here.

Response: we appreciate your meticulous review. We also got the statement was confusing and we have corrected and highlighted it in the“Discussion” section of the “Revised manuscript with track changes” Line 384-386, Page 14.

**Comment10:** Page 15: Re. this sentence, “Accordingly, receiving service from CRCtrained health care providers, having ANC visits, pregnancy planning status, giving birth during the daytime, and experiencing an obstetric complication were identified as determinants of RMC” : Again, this is incorrect and misleading. OB complication is NOT a determinant of RMC but a barrier to RMC or a risk factor for low RMC.

**Response:** thank you for the comment. you have raised the same comment in the above section (comment 8) and we have tried to address it one by one, and we kindly request you to check the responses here. As indicated by studies included in this SRMA, sustaining obstetric complication/s was/were identified as a negative determinant of RMC.

Comment11: Page 16: Re. the following sentence, “The possible justification could be that women who had adequate ANC visits had a better chance of acclimating to the health facility setup and developing close relations with the HCP. All of these are essential in ingraining trust in the facility's services, which resulted in RMC[39, 53]” please see my comment:

Since provider behaviors are the basis of RMC, an explanation that centers the change in provider behavior toward clients who had adequate ANC, or a change in the dynamic between provider and client might be mentioned here. Otherwise, is the hypothesis that women's attitudes or perceptions changed if they had adequate ANC? The WHO Bulletin definitions of D&A by Freedman et al might be interesting to consult here (<https://apps.who.int/iris/handle/10665/271621>).

**Response:** thank you very much for showing us the way to consider the other importance of having an adequate ANC on the provider side. We have incorporated that statement and evidence and highlighted them in the “Discussion” section of the “Revised manuscript with track changes” Line 426-428, Page 15.

**Comment12:** Page 17: Re. “This could be because women who experienced complications during labor are more likely to develop postpartum blues and depression, which can impede and lower the process and level of receiving RMC.” This statement needs a reference citation.

**Response:** Thank you for your suggestion. Accordingly, we added a reference for the statement and highlighted it in the “Discussion” section of the “Revised manuscript with track changes” Line 460, Page 16.

**Comment13:** Re. “In addition, complicated labor necessitates frequent and meticulous follow-up, which exhausts the provider and may result in service abandonment,” see my comment: There are a number of dimensions of D&A that could be associated with complicated birth theoretically (e.g., unconsented care, lack of information and choice, lack of respect for values and preferences, exclusion of companion of choice, lack of privacy...) therefore, this merits further discussion and literature search/citations.

**Response:** thank you for your insightful comment and suggestion. Accordingly, we have incorporated the other perspective and highlighted it in the “Discussion” section of the “Revised manuscript with track changes” Line 464-467, Page 17.

**__________________________________________________________________________________________________________END_______________________________________**

**THANK YOU!!!**

**Authors’ Response To Reviewer**#**2**

**General Comment:** This study gives very important results regarding the level and determinants of respectful maternity care during childbirth. However, in a few areas, here are my comments.

**Response:** Thank you very much for taking the time to review our work and for your positive feedback. We received your thoughtful, and generous review, along with helpful feedback and suggestions, as a valuable contribution to our ongoing work.

**Response to Revision comments or further considerations:**

**Comment1:** Why do you review the articles on RMC only from Ethiopia?

**Response:** thank you for the inquiry. We, all the authors agreed on the issue that knowing the national prevalence of RMC is researchable, and we conducted this SRMA. Studying and knowing the pooled prevalence will help policymakers and program planners build appropriate interventions to enhance the level of RMC, which is one of the four pillars of the health sector transformation plan(HSTP).

**Comment2:** The abstract is many worded, Abbreviations are used in the abstract section. need correction.

**Response:** those issues were also raised by an academic editor and we have corrected and highlighted throughout the “Abstract” section the “Revised manuscript with track changes”

**Comment3:** What is the unique characteristic of the quality of maternal and newborn health care? What is the level of quality care? The determinants of respectful maternity care are not mentioned clearly in the introduction section as mentioned in the results. (e.g., friendly care, timely care, discrimination-free care, and abuse-free care).

**Response:** Thank you for your question and suggestion. as per your suggestion, we have tried to incorporate the clear definition of RMC within its main components in the “Introduction” section of “Revised manuscript with track changes”, Line 85-88, Page 3

**Commnet4:** The topic can be refined ( or make short)

**Response:** thank you for the suggestion. We corrected it accordingly and highlighted it on the “Title page” of the “Revised manuscript with track changes”, Line 1-3, Page 1.

**Comment 5:** I think the population will be pregnant women. Reproductive age group is a vague term for RMC.

**Response:** we agree with your suggestion and we have corrected and highlighted it in the “Eligibility criteria” section of the “Revised manuscript with track changes” Line 140, Page5

**Comment 6:** Insufficient citation, particularly in discussion for safe interpretation.

**Response:** Thank you for your meticulous review. This issue was also raised by one of the reviewers and we have supported some the stataements in the discussion section with more updated articles.

**Comment 7:** Use correct tense, grammar, sentence, spelling, paraphrase, consistency…etc needs correction

**Response:** thank you for your meticulous review. We have checked all the issues your raised and we have tried to correct the issues accordingly.

__________________________________________________________________________________________________________END________________________________________

THANK YOU!!!

**Authors’ Response To Reviewer**#**3**

**General Comment:** Despite writing nicely I felt some issues in the manuscript. I suggest addressing these issues to accept for publishing it, My suggestions/ comments are as follows.

**Response:** Thank you very much for taking the time to review our work and for your positive feedback. We received your thoughtful, and generous review, along with helpful feedback and suggestions, as a valuable contribution to our ongoing work.

**Comment 1:** Abstract or summary section RMC measurement method is not clearly defined with specifying measurement scale (count, ordinal continuous, or binary). The study claimed the use of the random effect model to analyze 43 (some places 38) studies. Studies use a random effect model in panel data. This study evaluated mostly the results of cross section studies. If the studies were clustered, was that sample enough or statistical analysis? Missing full form of AOR.

**Response:** thank you for your insightful inquiry. As we mentioned in the manuscript the number of studies included in this meta-analysis were sixteen, not 43. All included studies were crossectional data with a binary response of receiving RMC or not. Due to a significant heterogeneity across the included studies, we used a random effect model with a DerSimonian Laird method, which is the most preferable one by many scholars. Regarding the sample size, all of the included studies had an adequate number of study participants to be included in the SRMA.

**Comment 2:** The writing of the introduction section is too long but it missed explaining vital things. In the last paragraph of the introduction section, the current knowledge of RMC requires further elaboration to justify. The message of the statement "the results have been inconsistent and varied" is inadequate.

**Response:** Thank you for the inquiry, this salient point has been noted and welcomed by the author team. Significant revisions have thus been made throughout the “introduction” section of the revised manuscript by removing some unnecessary and redundant statements. We also added some justification and highlighted it in the “introduction” section of the “Revised manuscript with track changes”, Line124-126, Page 4

**Comment3:** An illustration of the data screening process in the figure would make the paper more appealing to readers. Please refer to other meta-analysis-based papers in the health sector.

**Response:** We share your concern, but we used the most updated form of the PRISMA flow diagram for screening articles which was applicable for the majority of currently conducted studies in the health sector.

**Comment 4:** I suggest placing most figures and tables in the main body. Readers find it difficult to follow materials in supplements and appendix.

**Response:** thank you for your suggestion. But we were submitting those figures and supporting information as per the Plos One journal manuscript submission guideline. When the paper was accepted for publication, the production team will put all the figures as part of the main body of the article and it will be easy for the readers. This was the reason behind the submission of the figures independently.

**Comment 5:** Discussion: This study benchmarked with meta-studies of different countries. I am doubtful whether the dates of the publications are of similar times.

**Response:** Thank you for your meticulous review and insightful inquiry. We took into account the publication and study period of all the studies we used as references, that’s why we did not cite any studies published before 2012.

**Comment 6:** Hardly a few findings are generalized in the conclusion section. Most of the space is used for recommendations. I would avoid the strong word "should" to write recommendations.

**Response:** thank you for your valuable suggestion, that makes our conclusion more comprehensive for the readers. Thus, we added a statement showing the findings of subgroup analyses, and we highlighted it in the “Conclusion” section of the “Revised manuscript with track changes”, Line 480-481, Page 17.

__________________________________________________________________________________________________________END________________________________________

THANK YOU!!!

**Authors’ Response To Reviewer**#**4**

**General Comment:** The systematic review and meta-analysis on the determinants of RMC was good. It would be worthwhile for policymakers to plan and improve childbirth care. The following changes are required in this manuscript:

**Response:** Thank you very much for taking the time to review our work and for your positive feedback. We received your thoughtful, and generous review, along with helpful feedback and suggestions, as a valuable contribution to our ongoing work.

**Comment 1:** used the terms "prevalence" or "incidence" of RMC instead of "level of RMC"

**Response:** thank you for your insightful inquiry. We replaced it with the “prevalence” as per your suggestion throughout the revised manuscript.

**Comment 2:** Remove the last two lines of conclusion in the abstract: " A due empahsis.

**Response:** We remove it as per your suggestion.

**Comment3:** The introduction is too long, make it clear and to the point, focusing on research questions. I suggested including studies related to variables that affect RMC, the prevalence of RMC in Ethiopia health facilities or community-based facilities, and any differences in the prevalence of RMC in different sectors.

**Response:** Thank you for the inquiry, this salient point has been noted and welcomed by

the author team. Significant revisions have thus been made throughout the “introduction” section of the revised manuscript by removing some unnecessary and redundant statements. As per your valuable suggestion, we incorporated the prevalence (i.e range) and factors affecting the receipt of RMC in the “Introduction” section of the “Revised manuscript with track changes”, Line111-114, Page 4

**Comment 4:** Study selection process: include how many articles for SR and meta-analysis.

**Response:** thank you for your suggestion. We added the statement and highlighted it in the “Study selection process” section of the “Revised manuscript with track changes”, Line185-186, Page 6

**Comment 5:** Incusion criteria: study design: make clear regarding reporting the level of RMC, I suggested replacing the Prevalence of RMC throughout the study by prevalence.

**Response:** Thank you for your meticulous review and insightful inquiry. We corrected it accordingly throughout the revised manuscript.

**Comment 6:** Could you explain which threshold of p-value has been used for statistical significance when using the Cochrane Q test to determine statistical heterogeneity?

**Response:** thank you for the inquiry. We used a p-value less than 0.05

**Comment 7:** write components of RMC instead of domains of RMC. explain its details in the results section.

**Response:** Thank you for your suggestion and we amend it accordingly and highlighted in “The prevalence of RMC in Ethiopia” section of the “Revised manuscript with track changes”, Line 274, Page 10

**Comment 8:** Explain sensitivity analysis based on..(Fig 6); elaborate on this information in the result section.

**Response:** We discussed and concluded the findings of the sensitivity analysis in the “Sensitivity analysis” section of the “Revised manuscript with track changes”, Line 306-308, Page 12

**Comment 9:** The discussion was so long. Could you please focus on the main objective of the study? Look for the first paragraph of the conversation (you can make it very brief).

**Response:** we appreciate your meticulous review. Despite most of the findings of the study needing wider discussion and explanations, we tried to shorten the discussion section by omitting some redundant and vague statements.

**Comment 10:** Could you include some other critical factors that influence components of RMC or the overall prevalence of RMC?

**Response:** Thank you for asking. While we run the meta-analysis, only six variables were identified as significant determinants of RMC.

**Comment 11**: Include the current study's strength in the last paragraph before the limitation.

**Response:** Thank you for the suggestion, we do accordingly and highlighted it in the “Discussion” section of the “Revised manuscript with track changes”, Line 468-469, Page 16

**Comment 12:** _Provide references in discussions ection line start...On the other hand, the tendency to receive low RMC during the night shift may be explained by the low staff number -to- obstetric cases -Provide reference for line start..... In addition, complicated labor necessitates frequent and meticulous follow-up, which exhausts the provider and may result in service abandonment

**Response:** thank you for your kind suggestion which is helpful to enhance the scientific plausibility of the findings. Accordingly, we cited relevant studies that support the justifications behind the association. The citations were highlighted in the “Discussion” section of the “Revised manuscript with track changes”, Line 451 and 454, Page 16

Comment 13: Include some information regarding the strength of evidence and write some of the geographical differences in RMC prevalence in Ethiopia.

Response: we appreciate your meticulous review. As per suggestion, we added a statement that deals with a sub-group analysis and highlighted it in the “Conclusion” section of the “Revised manuscript with track changes”, Line 480-481, Page 17

__________________________________________________________________________________________________________END________________________________________

THANK YOU!!!
