# Supplementary material for: The prevalence of respectful maternity care during childbirth and its determinants in Ethiopia: A systematic review and meta-analysis
Source: PLoS One. 2022 Nov 23;17(11):e0277889. doi: 10.1371/journal.pone.0277889 (PMC9683616; doi:10.1371/journal.pone.0277889)
Supplement: Attachment — Submitted filename: Response to Reviewers.docx [file pone.0277889.s008.docx]

**AUTHORS’ RESPONSE TO ACADEMIC EDITOR**

1. **Author’s Response to Academic Editor**

**Dear: Zemenu Yohannes Kassa, Msc (Academic Editor)**

We thank you for a thorough reading and constructive comments and suggestions on our manuscript and for the opportunity to revise and resubmit. We are pleased to submit the revised research article “**The prevalence of respectful maternity care during childbirth and its determinants in Ethiopia: A systematic review and Meta-analysis**” for your consideration in the special collection of Plos One. On the following pages, you will find our responses to the questions raised from your side and two reviewers' comments. On behalf of my co-authors, I thank you for your consideration of this resubmission. We appreciate your time and look forward to your response.

Sincerely,

Aklilu Habte(MPH)(corresponding author)

**[aklilihabte57@gmail.com](mailto:aklilihabte57@gmail.com)**

**Dear Dr. Habte,**

Thank you for submitting your manuscript to PLOS ONE. After careful consideration, we feel that it has merit but does not fully meet PLOS ONE’s publication criteria as it currently stands. Therefore, we invite you to submit a revised version of the manuscript that addresses the points raised during the review process.

**General comment and suggestion**

I assessed your revision as Editor I am pleased to inform you that it is potentially publishable in PLOS ONE and I invite authors to perform the required minor revisions.

**Response:** I thank you for the time and effort made to review the manuscript in detail, for your constructive comments and for the opportunity to revise and resubmit. After completion of the suggested edits, the revised manuscript has benefitted from an improvement in the overall presentation and clarity. We have have highlighted the document to indicate any changes in word, phrase, or sentences.

**Comment 1:** In the title, you should remove contemporaneous.

**Response:** We have amended the title as per your suggestion and highlighted it on the “title page” of the “Revised manuscript with track changes” Line 2-3, Page1.

**Comment 2:** line 49 rewrite again,line 287,288,325,337, 366, 384, 460 and 470 grammar error.

**Response**: thank you for your comment. As per your suggestion, we have tried to correct those sentences in the above mentioned lines. All the corrected versions were highlighted throughout the revised version of the manuscript.

_____________________________END________________________________________

THANK YOU!!!
